# Real-world outcomes of Deep Brain Stimulation for dystonia treatment: Protocol for a prospective, multicenter, international registry

**DOI:** 10.1371/journal.pone.0303381

**Published:** 2024-09-27

**Authors:** Alberto Albanese, Roshini Jain, Joachim K. Krauss

**Affiliations:** 1 Department of Neurology, IRCCS Humanitas Research Hospital, Rozzano, Milano, Italy; 2 Boston Scientific Neuromodulation, Valencia, CA, United States of America; 3 Department of Neurosurgery, Hannover Medical School, Hannover, Germany; IRCCS Medea: Istituto di Ricovero e Cura a Carattere Scientifico Eugenio Medea, ITALY

## Abstract

**Introduction:**

Deep Brain Stimulation (DBS) is an established therapeutic approach for the treatment of dystonia. However, to date, no large-scale or comprehensive DBS dystonia patient registry has been yet undertaken. Here, we describe the protocol for a world-wide registry of clinical outcomes in dystonia patients implanted with DBS.

**Methods and analysis:**

This protocol describes a multicenter, international clinical outcomes registry consisting of up to 200 prospectively enrolled participants at up to 40 different sites to be implanted with a constant-current, multiple independent current controlled (MICC) DBS device (Vercise DBS Systems, Boston Scientific) for treatment of dystonia. Key inclusion criteria for registry candidates include the following: understanding of study requirements and treatment procedures, a signed written informed consent form prior to participation, and meeting all criteria established in the locally applicable Instructions for Use (IFU) for the implanted DBS system. Key clinical endpoints include (but are not limited to) the evaluation of disease state (Burke-Fahn-Marsden Dystonia Rating Scale [BFMDRS], Toronto Western Spasmodic Torticollis Rating Scale (TWSTRS), quality of life (Short Form Health Survey-36, Short Form Health Survey-10), and treatment satisfaction (Clinical Global Impression of Change [CGI-Clinician; CGI-Subject; CGI-Caregiver]) at 6-months, 12-months, 2-years, and 3-years post-lead placement. Adverse events are documented and reported using structured questionnaires.

**Perspectives:**

Treatment of patients with dystonia using DBS has progressed considering recent technological advances. This international dystonia outcomes registry aims to collect and evaluate real-world clinical data derived from patients who have been implanted with a constant-current, MICC-equipped DBS system (with available directional capabilities), per standard of care.

## Introduction

Dystonia is a movement disorder characterized by involuntary muscle contractions causing abnormal, often repetitive, movements, postures, or both. Dystonic movements are typically patterned and twisting and may be tremulous [1]. The classifications of dystonia are based on two axes: Axis 1 describes patient clinical features, and Axis 2 is defined by etiology. Patient clinical features considered by the current classification are age at onset, body distribution, temporal pattern, and associated features (whether dystonia occurs in isolation, in combination with other movement disorders or with systemic features) [1]. Deep brain stimulation (DBS) is commonly used in the management of symptoms from movement disorders including Parkinson’s disease, essential tremor and dystonia [2–8]. Over the last decade, technological advances have provided new strategies to help improve patient outcomes and the overall experience with DBS-based therapy. These include the introduction of multiple-source constant-current devices, segmented leads, increased battery longevity, and automated optimization of DBS programming [9–12]. When treating dystonia, clinical responses to DBS vary across patients (inter-patient) and within patients (intra-patient) over time, indicating the need for tools designed to detect such changes [13].

The drive to improve the quality of patient care, especially amidst an ever-evolving technological landscape, has led to the implementation of large medical device registries designed to track and assess clinical outcomes, safety, cost-effectiveness, and other aspects pertinent to the evaluation of the delivery of healthcare (e.g., procedural assessment, disease burden, research, other) [14–16]. Registries, by definition, prospectively collect data obtained from real-world clinical settings so as to surveil outcomes derived from within the typical conditions experienced by a specific patient population of interest. Notably, published data generated from registries are increasingly utilized not only by practicing clinicians, but also by other key decision- and policymakers within institutional frameworks such as regulatory bodies, public health advocates, and healthcare payors [14–17]. Registries have the additional advantage to facilitate standardization of practices among different centers. However, to our knowledge, only very few registries tracking outcomes of DBS in dystonia patients have been implemented, and these mainly were designed to specifically focus on tracking pediatric cases [18, 19]. As such, we have now embarked on the initiation of the first global-wide registry of clinical outcomes in patients implanted with a DBS device for use in the treatment of medically refractory dystonia. The registry endeavors to bridge the existing gap in disease management, treatment effectiveness, health economic value and patient outcomes. Here, we describe the protocol of this prospective, multicenter, international DBS patient outcomes registry.

## Materials and methods

### Study design

The Vercise Deep Brain Stimulation (DBS) Dystonia Registry is a prospective, multicenter, international registry designed to compile real-world clinical outcomes with the use of commercially approved DBS systems (Vercise, Boston Scientific) for treatment of dystonia per local instructions for use (IFU). The registry (identified as NCT02686125 and study identification #A4012 [version AD] on Clinicaltrials.gov) will prospectively enroll up to 200 participants at up to 40 different international sites. Study participants will be followed up for 3 years after first lead placement. Participation in the registry is completely voluntary. The registry is being conducted in accordance with ISO 14155: Clinical Investigation of Medical Devices for Human Subjects–Good Clinical Practice, Declaration of Helsinki, and pertinent individual country laws and regulations. The registry protocol (study #A4012) has obtained Ethics Committee (EC) approval in the following countries: Belgium (Committee on Medical Ethics at UZ Ghent [ref. B670201940456; July 23, 2019]), Germany (Medical Ethics committees at the Julius Maximilian University of Würzburg [ref. 9/16_z; September 6, 2016], Hamburg Medical Association [ref. MC-335/15; April 20, 2016], Medical School of the Christian Albrecht University in Kiel [ref. B 313/17; November 20, 2017], Medical School of Hannover [ref. 2782–2015; August 3, 2015], Heinrich Heine University Düsseldorf [ref. 2015114563; March 3, 2016], Westphalian-Lipe Medical Association and the Westphalian-Wilhelms University Münster [ref. 2016-253-b-S; June 8, 2016], Medical Faculty of the University of Cologne [ref. 15–367; December 23, 2015], Philipps University of Marburg [ref. 78/21; June 4, 2021], State Medical Association Rheinland [ref. 2020–14973_1; June 17, 2020], and University Hospital Freiburg [ref. 424/19; October 24, 2019]), Hungary (Ogyei, National Institute of Pharmacy and Nutrition [ref. OGYEl/32313-4/2018; December 31, 2021]), Israel (Helsinki Committee [ref. 0609-19-HMO; April 25, 2021]), Italy (Ethics Committees at the University Agostino Gemelli IRCCS, Catholic University of the Sacred Heart [ref. 0033604/19; July 29, 2019], University Hospital of Ferrara [ref. 150895; July 14, 2016], and Autonomous Region of Friuli Venezia Giulia [ref. 8833; March 14, 2017]), Netherlands (METC Leiden-Den Haag-Delft [ref. Z19.061; July 3, 2020]), Poland (Bioethics Committee at the University of Warmia and Mazury in Olsztyn [ref. 14/2018; April 12, 2018]), Russia (Ethic Committee at the N.N. Burdenko National Medical Research Center of Neurosurgery [ref. 3957; May 18, 2019]), Republic of Korea (Asan Medical Center Institutional Review Board [ref. 2021–0380; January 26, 2022], The Catholic University of Korea, Incheon St. Mary’s Hospital Institutional Review Board [ref. 2019-3028-0003; November 16^th^, 2020]), Spain (Ethics Committees of the Principality of Asturias, University Hospital [ref. 10/15; November 26, 2015], University Hospital Ramón y Cajal [ref. SIN-EU-001; January 25, 2016], and University Hospital Virgen de la Arrixaca [ref. AC 24/09/15; November 30, 2015]), and United Kingdom (Research Ethics Service Committee South Central, Oxford C [ref. 15/SC/0293; July 20, 2015]).

**Fig 1:**
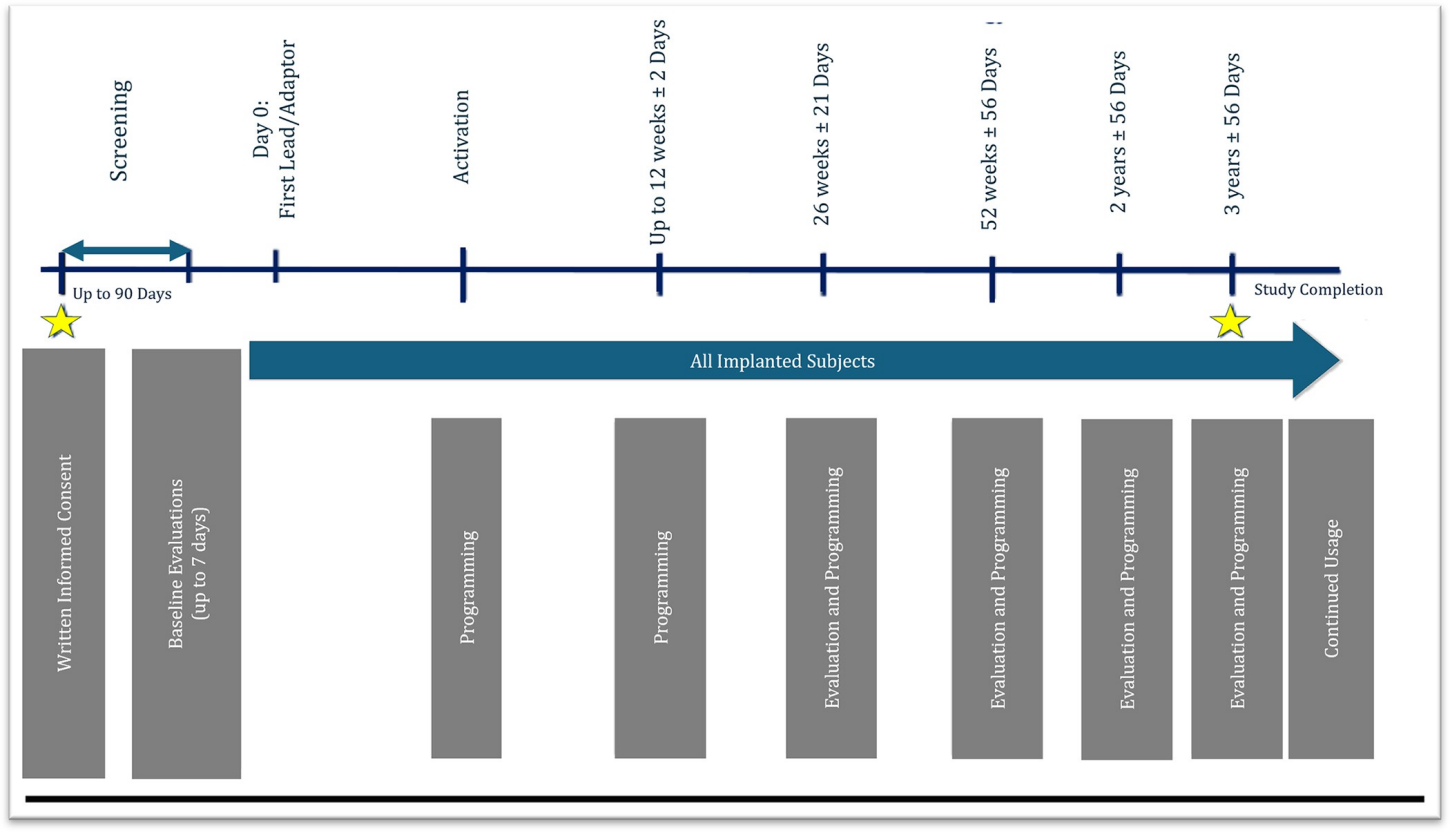
Dystonia registry study timetable.

Study candidates undergo screening for up to 90 days during which their eligibility for inclusion is determined. Upon confirmation of eligibility, participants receive the DBS implant as part of their standard of care (Day 0). Following completion of implant, therapy is activated, and participants return to the clinic for their follow-up study visits as shown in Fig 1 and Table 1. Study participants complete assessments/questionnaires according to the type of dystonia they are diagnosed with at the following timepoints: 6-months, 12-months, 2-, and 3-years post-lead placement. Adverse events (AEs) are collected.

**Table 1:** Clinical activities included in study protocol.

			STUDY PERIOD						
	Enrollment	Screening	Implantation	Activation	Post-Allocation				Close-out
TIMEPOINT	≤90 days	≤7 days	Day 0	*	6 mos. ±21 days	1 yr. ±56 days	2 yrs. ±56 days	3 yrs. ±56 days	˃3 yrs.
**Eligibility screen**	X								
**Informed consent**	X								
**Baseline Evaluation**		X							
**1**^**st**^ **Lead/Adaptor**			X						
**DBS Programming**				X					
**Evaluation of DBS Efficacy and Safety; Programming**					X	X	X	X	
**Continued Usage**									X

mos. = month

yr. = year

yrs. = years

*note = no set timepoint is required for Activation, this is determined by each site, per standard of care

All participating investigational centers in the registry will provide to the sponsor documentation verifying that corresponding Ethics Committee (EC) registration has been submitted to the appropriate agency, as applicable according to national/regulatory requirements. A copy of the written EC and/or competent authority approval of the protocol (or permission to conduct the study) and Informed Consent Form (ICF), must be received by the sponsor before recruitment of subjects into the study and shipment of investigational product/equipment. Annual EC approval and renewals will be obtained throughout the duration of the registry as required by local/country or EC requirements. Informed consent to participate is required to be in writing. To be enrolled in the registry, candidates must complete, sign and date a written informed consent form (ICF); for patients between the ages of 7–18 years old, parent or guardian consent is required consisting of a dated signature. The context of the registry must be fully explained, and prospective registry participants (and/or parent/guardian for those between the ages of 7–18 years old) must be given the opportunity to ask questions and have those questions answered to their satisfaction.

This consenting process is documented in each subject’s source data, as per EC approval. In accordance with SPIRIT reporting guidelines, appropriate information relevant to the Vercise Deep Brain Stimulation (DBS) Dystonia Registry in herein described within this summary of the approved protocol [20].

### Inclusion and exclusion criteria

Study participants are required to meet all inclusion criteria and have none of the exclusion criteria to participate in the study (Table 2). Inclusion criteria include the following: (1) understands the study requirements and treatment procedures, and provides written informed consent before any study-specific tests or procedures are performed; (2) plans to receive a currently available DBS system (Boston Scientific) and any other components (e.g., leads, extension, pocket adaptor) compatible with the DBS system (Boston Scientific); (3) meets criteria established in the locally applicable Directions for Use (DFU) for the DBS system (Vercise) for treatment of dystonia; and (4) at least 7 years old (parent or guardian consent is required in patients who are younger than 18 years at the time of consent). Participants who meet any contraindication for the DBS system (Boston Scientific) per locally applicable DFU are excluded from the study.

**Table 2:** Inclusion/exclusion criteria.

Candidates who meet all of the inclusion criteria may be given consideration for participation in the registry, provided no exclusion criterion is met.	
Inclusion Criteria	• Understands the study requirements and the treatment procedures and provides written informed consent before any study-specific tests or procedures are performed • Receive a currently available Vercise DBS System and any new CE marked components (i.e., leads, extension, Pocket Adaptor, CP, etc.) compatible with the Vercise System • Meets criteria established in the locally applicable Vercise DBS System Directions for Use (DFU) for Dystonia • At least 7 years old. Parent or guardian consent is required in patients who are younger than 18 years at the time of consent
Exclusion Criteria	• Meets any contraindication in the Vercise DBS System locally applicable Directions for Use

### Clinical outcomes

Several clinical endpoints related to disease state, quality-of-life, and satisfaction are included in the registry. During the Baseline visit, a detailed evaluation of disease state and its impact on health is performed (e.g., medical history, concomitant medications). A detailed history to characterize the study participant’s dystonia and any other relevant surgeries and chronic conditions with specific attention to the following is conducted: intracranial surgery, stroke, seizure; history of prior neuromodulation implants; psychiatric history; presence of fixed joint contractures and cervical myelopathy; reaction to materials (such as latex, metals) and history of foreign body reactions. Additionally, an assessment of the type of dystonia (idiopathic, inherited or acquired), region of involvement (e.g., hand or cervical) and body distribution is collected. Genetic testing, if available, is documented per standard of care.

Clinical endpoints are collected at Baseline, 6-months, 12-months, 2-years, and 3-years following first lead placement. Based on diagnosed dystonia sub-type (i.e., including cervical component), assessments are administered to study participants. These include the following: Burke-Fahn-Marsden Dystonia Rating Scale (BFMDRS), Clinical Global Impression of Change (CGI-Clinician; CGI-Subject; CGI-Caregiver), Global Dystonia Rating Scale (GDS), Montreal Cognitive Assessment (MoCA), Satisfaction with Treatment, Short Form Health Survey-36 (SF-36v2), Short Form Health Survey-10 (SF-10v2), Short Form Health Survey for Children (as applicable) and the Toronto Western Spasmodic Torticollis Rating Scale (TWSTRS) [21–27]. Additionally, change in economic value and total cost of treatment and resource utilization (RUI) including hospital visits, emergency room (ER) visits, outpatient visits, home care, physician visits by specialty, are tracked and assessed throughout the course of the registry. All prescribed dystonia-related medications are also collected. A summary schedule of clinical assessments is provided in Table 3. For exploratory purposes only, sub-group analyses may be conducted per the availability of the following types of data: age at surgery, age of disease onset, duration of disease, dystonia classification, genetic status, STN versus GPi implanted subjects.

**Table 3:** Data collection schedule.

Study assessment*	Screening	Baseline	Implant	Activation	6-months	1, 2, 3 Years
Informed Consent§	X					
Inclusion/Exclusion Criteria Evaluation	X					
Adverse Event (AE)†		X	X	X	X	X
Burke-Fahn-Marsden Dystonia Rating Scale (BFMDRS)^¶^		X			X	X
Clinical Global Impression of Change (CGI-C: Clinician)					X	X
Clinical Global Impression of Change (CGI-C: Subject)					X	X
Clinical Global Impression of Change (CGI-C: Caregiver)					O	O
Global Dystonia Rating Scale (GDS)		X			X	X
Montreal Cognitive Assessment (MoCA)		X			X	X
Resource Utilization Inventory (RUI)		X			X	X
Satisfaction with Treatment (SWT)					X	X
Short Form Health Survey-10 (SF-10v2)^Δ^		X			X	X
Short Form Health Survey-36 (SF-36v2)^μ^		X			X	X
Toronto Western Spasmodic Torticollis Rating Scale (TWSTRS)°		X			X	X

* Assessments and questionnaires are utilized based on subject classification and age

^¶^ only subjects diagnosed with non-cervical dystonia

^Δ^ subjects aged 18 years and older at time of consent

^μ^ subjects under the age of 18 years at time of consent

° subjects with cervical dystonia only (18 years and older at time of consent)

† Only adverse events related to the device, stimulation, or procedure, and serious adverse events will be recorded

§ In subjects younger than 18 years of age, form to be completed by both registry participant and the parent/guardian.

X = required; O = optional

### Safety outcomes

Adverse events are collected for all registry participants. The rates of occurrence of all serious adverse events (SAEs), and all adverse device effects including serious adverse device effects (SADEs) and unanticipated serious adverse device effects (USADEs), are recorded, and evaluated throughout the registry up to 3 years following first lead placement. Adverse events occurring during participation in the registry will be classified according to seriousness, severity, and relationship to either study procedure and/or study-device action. Furthermore, an assessment of actions taken to address adverse events and their outcome will be conducted. The potential relationship of all adverse events to the study device, study procedures and/or stimulation will also be completed by study investigators (i.e., Unlikely, Possibly, Probably or Causally Related). Study investigators will report all Device Deficiencies, Unanticipated Adverse Device Effects/Unanticipated Serious Adverse Device Effects, and new findings/updates in relation to already reported events. Underlying diseases are not reported as AEs unless there is an increase in severity of frequency during the course of the registry. There are anticipated risks that may be associated with the use of a DBS System and are made available as part of Informed Consent and patient manuals.

To promote early detection of safety issues, the sponsor will provide evaluations of safety events. During regularly scheduled monitoring visits, clinical research monitors will support the dynamic reporting process through their review of source document information.

If a protocol revision is necessary which affects the rights, safety or welfare of enrolled registry participants or scientific integrity of the data, an amendment to the registry protocol will be undertaken. As such, appropriate Ethics Committee approvals of the revised protocol must be obtained prior to implementation.

### Device and procedures

Study participants are implanted with the Vercise^TM^ DBS System (Boston Scientific) as available in the patient’s country of origin. The family of Vercise DBS systems (Boston Scientific) is comprised of primary cell and rechargeable implantable stimulators, and DBS leads (including directional leads). The system is designed to precisely deliver current to targets using a multiple-source, constant-current technology known as Multiple Independent Current Control (MICC) enabling fractionalization of both cathodic and anodic current within activated lead contacts and between lead contacts and the IPG. The DBS leads can be used for unilateral or bilateral implantation/stimulation; extensions are used to connect leads with the IPG subcutaneously implanted below the clavicle. The DBS leads are typically implanted in the globus pallidus internus (GPi) but may also be implanted in the subthalamic nucleus (STN). Depending on the type of lead implanted, leads can deliver stimulation exclusively in a ring mode (around the lead) or if a directional lead is used in a ring or directional mode (away from the lead). Once leads are implanted, they are secured using physician’s standard lead fixation technique. A SureTek™ Burr Hole Cover may be used for securing the lead to the skull. Participants receive a remote control and a charging system, as applicable.

### Statistical analysis

All clinical endpoints will be summarized using descriptive statistics for continuous variables (e.g., mean, standard deviation, N, minimum, maximum) and frequency tables or proportions for discrete variables. Estimates of all endpoints will be reported, as well as the 95% confidence intervals. Due to the nature of the registry, pre-specified hypothesis/analysis will not be completed. Analyses will be performed using data pooled across institutions. Multivariate analysis techniques, including contingency tables and logistic regression for binary outcomes and analysis of variance for continuous measures, will be used to assess differences among study institutions to justify pooling data across institutions. Multivariate models will be used to assess the predictability of outcome from the risk factors. Interim analysis will proceed when sufficient power is reached. No pooling of data will be performed per any sub-groups identified and assessed in the registry. In the safety analysis, all subjects who sign the IRB/EC-approved written Informed Consent form will be included. Changes from the planned statistical methods after performing analyses will be documented along with a basis for the deviation.

### Patient selection

Selection of patients are made from the study investigator’s usual patient population, including referred patients. All patients meeting the inclusion/exclusion criteria and having signed the Informed Consent Form will be eligible for participation in the study. Consecutively eligible patients will be enrolled in the study, minimizing selection bias.

### Data management

All source documentation will be retained at each site participating in the registry. Examples of source documents include, but are not limited, to the following: informed consent form and consent process documentation; documentation of medical history components; medical records from clinical visits (e.g., progress notes) and operative procedures; study-specific source document worksheets (if applicable). Subject data will be recorded in a limited access secure server designed to meet regulatory compliance for deployment as part of a validated system compliant with laws and regulations applicable to the conduct of clinical studies pertaining to the use of electronic records and signatures. All participating clinical investigators and/or participating sites will maintain, at the investigative site and in original format all essential study documents and source documentation that support the data collected on the study subjects in compliance with International Conference on Harmonization (ICH)/Good Clinical Practice (GCP) guidelines. Additionally, data sharing agreements were established with the sponsor prior to study start.

All information and data sent to the sponsor concerning participants in the registry will be considered confidential by the sponsor. Only authorized sponsor personnel or a sponsor representative will have access to confidential records. As such, the sponsor will keep health information of those subjects participating in the registry confidential in accordance with all applicable laws and regulations. Information received during the study will not be used to market to subjects participating in the registry and names will not be placed on any mailing lists or sold to anyone for marketing purposes. Dissemination of study results in the form of peer-reviewed journal publication or other publications will adhere to the requirements of the International Committee of Medical Journal Editors (ICMJE).

Monitoring will be performed during the study to assess continued compliance with the protocol and applicable regulations. In addition, the monitor verifies that study records are adequately maintained, that data are reported in a satisfactory manner with respect to timeliness, adequacy, and accuracy, and that the study site continues to have sufficient staff and facilities to conduct the study safely and effectively. The investigator/institutional study site must provide direct access to original source documents by sponsor personnel, their designees, and appropriate regulatory authorities. Monitoring visits will be conducted at regular intervals during the clinical investigation to assess the continued acceptability of the facilities, the continued compliance with applicable regulations and the maintenance of adequate study records.

The sponsor reserves the right to terminate the study at any stage but intends to exercise this right only for valid scientific or administrative reasons and reasons related to protection of participating subjects. Investigators, associated EC, and/or regulatory authorities, as applicable, will be notified in writing in the event of study termination.

## Discussion

Clinical patient registries can provide substantial value given their capacity to collect several types of real-world data from a heterogenous collection of participating sites pertaining to a specific patient cohort of interest. Large-scale, multicenter registries have been shown to enable the advancement of new collaborative research initiatives and standardized disease management algorithms as well as identification of gaps in treatment and/or data collection approaches [18, 28. 29]. It therefore is of particular importance that such registries be conducted amongst patient populations for which there is an evolving understanding of how best to diagnose, treat, and care for serious disease.

The Vercise Deep Brain Stimulation (DBS) Dystonia Registry, as described here in this protocol summary, represents the first comprehensive, large-scale, international collection of real-world clinical and safety outcomes in patients implanted with a multiple independent constant-current, (MICC-equipped) DBS device for use in the treatment of dystonia. DBS has been used since the 1980s for the treatment of motor symptoms associated with Parkinson’s disease, and it was introduced for the treatment of dystonia in the 1990s [7, 30–32]. The FDA approval to use DBS as a Humanitarian Exemption for treatment of dystonia occurred in 2003 [32]. Many studies have since measured the risks and benefits associated with this type of treatment for dystonia, and considerable reduction in symptoms has been observed [12, 32–40].

The use of a constant-current, MICC-equipped DBS system allows for a wider range of stimulation parameters and finer control of stimulation (as opposed to traditional constant-voltage DBS, or single source constant current devices). This is important given that dystonia patients tend to achieve maximum relief when the applied electrical field(s) is/are accurately localized to the appropriate target(s) [41]. In addition, improper placement of DBS leads in dystonia patients has been demonstrated to contribute greatly to sub-optimal outcomes [42]. Therefore, appropriate selection, localization, and utilization of lead electrodes according to pre-surgical planning and/or available imaging or other tools (e.g., software algorithms) is key when treating dystonia patients successfully using DBS. As such, all registry participants are implanted with a DBS system designed with multiple sources of current each source designated for an available lead contact and one for the IPG case (i.e., MICC), thereby permitting the fractionalization and adjustment of cathodic and anodic current, and in turn promoting improved field targeting and hence optimization of the total Volume of Tissue Activated (VTA). Study participants will receive standard or directional leads as per standard of care. With directional leads, the orientation and steering of applied electrical fields can be adjusted with enhanced flexibility (versus more traditional omnidirectional leads) as needed per patient. Since the early experience with a fully implantable directional system, the ability to directionally steer stimulation has been shown to facilitate expansion of therapeutic window (i.e., the difference in amplitude between the current necessary to induce a therapeutic response versus that which elicits an side effect) [43–45]. Given the current pace of development of various technologies such as closed loop neuromodulation, novel stimulation targets, stimulation waveforms, along with the mounting acquisition of knowledge and understanding of brain function and disorders, the need to collect real-world evidence is paramount [46].

There are limitations in the design of large, multicenter patient registries. Registry data collected solely in an observational manner, are accordingly only at-best descriptive in nature, and thus it is typically not possible to discern the exact cause(s) of any unanticipated outcomes. While it may be possible to develop generalizable inferences and hypotheses from registry data, with regard to the likely real-world outcomes and experiences of dystonia patients using DBS, it is not appropriate to use observationally collected data to derive methodologies for use in predicting outcomes in particular patients. Additionally, there is a risk of loss to follow-up or attrition in registries that span over long durations in time. However, this prospective, multicenter, international registry will collect real-world data up to 3-years post-DBS implant providing key insights related to clinical effectiveness, safety, and overall patient experience.

### Summary

This prospective multicenter registry is aimed to contribute to the available clinical evidence for the treatment of dystonia and will provide valuable insights on the use of DBS when utilized in the real-world clinical setting, per standard or care.

## Supporting information

S1 Checklist SPIRIT checklist for *Trials*. (DOCX)

S1 File (PDF)
