# Supplementary material for: Real-world outcomes of Deep Brain Stimulation for dystonia treatment: Protocol for a prospective, multicenter, international registry
Source: PLoS One. 2024 Sep 27;19(9):e0303381. doi: 10.1371/journal.pone.0303381 (PMC11432838; doi:10.1371/journal.pone.0303381)
Supplement: S1 File — (PDF) [file pone.0303381.s002.pdf]

Registry of Deep Brain Stimulation with the VERCISE™ System for treatment of Dystonia:

Vercise DBS Dystonia Registry

CLINICAL PROTOCOL

Sponsored By

Boston Scientific Corporation

Neuromodulation

25155 Rye Canyon Loop

Valencia, CA 91355

United States of America

International Representative

Boston Scientific Limited

Ballybrit Business Park,

Galway, Ireland

2. Protocol Synopsis

Registry of Deep Brain Stimulation with the VERCISE™ System for treatment of Dystonia:<br>Vercise DBS Dystonia Registry	
**Objectives**	To compile characteristics of real-world outcomes, economic value and technical performance of Boston Scientific Corporation's commercially approved Vercise^{TM} Deep Brain Stimulation (DBS) System, when used according to applicable Directions for Use (DFU)
**Planned Indication(s) for Use**	The Vercise™ Deep Brain Stimulation System is indicated for use in unilateral or bilateral stimulation of internal globus pallidus (GPi) or the subthalamic nucleus (STN) for treatment of intractable primary and secondary dystonia, for persons 7 years of age and older.
**Test Device**	Boston Scientific Corporation Vercise^{TM} System A neurostimulation system consisting of an implantable pulse generator (IPG), DBS leads, DBS extensions, Pocket Adaptor, Burr Hole Covers, surgical tools, and external devices (programming system, Remote Control, and Charging system).
**Study Design**	Prospective, on-label, multi-center, international registry
**Planned Number of Subjects**	Up to 200 subjects
**Planned Number of Centers / Countries**	up to 40 international sites
**Study Assessments**	The following assessments will be conducted in dystonia patients based on their dystonia sub-group, classification, and age. These assessments will be used to derive the study endpoints as applicable (in alphabetical order):<br>• Burke-Fahn-Marsden Dystonia Rating Scale

	(BFMDRS);<br>• Clinical Global Impression of Change (CGI-C);<br>• Clinical Global Impression of Change – Subject (CGI-C: Sub);<br>• Clinical Global Impression of Change – Caregiver (CGI-C: Crg); OPTIONAL<br>• Global Dystonia Scale (GDS);<br>• Montreal Cognitive Assessment (MoCA)<br>• Resource Utilization Inventory (RUI);<br>• Satisfaction with Treatment (SWT);<br>• SF-36v2 Health Survey in subjects age 18 and over at the time of consent, and SF-10v2 Health Survey in subjects under the age of 18 years at the time of consent;<br>• Toronto Western Spasmodic Torticollis Rating Scale (TWSTRS).
**Clinical Endpoints**	The following clinical endpoints will be analyzed for each sub-group of dystonia separately (Primary vs. Secondary):<br>• Proportion of subjects with 30% or greater reduction in Baseline BFMDRS or TWSTRS score at 26 weeks, 52 weeks, 2 years, and 3 years post first lead placement<br>• Change in BFMDRS scores from Baseline to 26 weeks, 52 weeks, 2 years, and 3 years post first lead placement<br>• Change in TWSTRS scores from Baseline to 26 weeks, 52 weeks, 2 years, and 3 years post first lead placement<br>• Change in SF-36v2 score (SF-10v2 in patients under the age of 18 years at the time of consent) from Baseline to 26

	weeks, 52 weeks, 2 years, and 3 years post first lead placement<br>• Change in GDS scores from Baseline to 26 weeks, 52 weeks, 2 years, and 3 years post first lead placement<br>• Clinical Global Impression of Change (CGI-C) rating score, as assessed by neurologist, at 26 weeks, 52 weeks, 2 years, and 3 years post first lead placement<br>• Clinical Global Impression of Change – Subject (CGI-C: Sub), as assessed by subject, at 26 weeks, 52 weeks, 2 years, and 3 years post first lead placement<br>• Clinical Global Impression of Change – Caregiver (CGI-C: Crg), as assessed by caregiver, at 26 weeks, 52 weeks, 2 years, and 3 years post first lead placement
**Safety Parameters**	Rates of occurrence of all serious adverse events (SAEs) and all adverse device effects (ADEs), including serious adverse device effects (SADEs) and unanticipated serious adverse device effects (USADEs) at 3 years post first lead placement.
**Health Economics Endpoints**	• Total cost of treatment and resource utilization from Baseline through end of study (RUI)<br>• Change in economic value from Baseline to 26 weeks, 52 weeks, 2 years and 3 years post first lead placement.
**Study schedule**	**Screening Period: Eligibility determination**<br>• Up to 90 days prior to first lead implant or IPG placement if the Pocket Adaptor is used.<br>**Baseline visit**<br>• Assessments prior to implant procedures.

	**Staged Implants: Device Placement**<br>• Day 0 is the date of the first lead implant procedure or IPG placement if the Pocket Adaptor is used.<br>• Typically 1 to 3 procedures per site's preferred practice.<br>**Activation**<br>• Programming/Device activation<br>**Programming and Evaluation visits**<br>• Up to Week 12 visit (±21 days) (programming only)<br>• Week 26 visit (±21 days)<br>• Week 52 visit (±56 days)<br>• Year 2 visit (±56 days)<br>**End of Study visit: Programming and Evaluation visit**<br>• Year 3 visit (±56 days)<br>Subjects may come in as needed for programming and/or evaluation of adverse events during the study.
**Study Duration**	The study will be considered complete after all subjects complete their year 3 visit
**Inclusion Criteria**	IC1. Understands the study requirements and the treatment procedures and provides written informed consent before any study-specific tests or procedures are performed.<br>IC2. Receive currently available Vercise System and any new CE marked components (i.e., leads, extension, Pocket Adaptor, CP, etc.) compatible with the Vercise System<br>IC3. Meets criteria established in the locally applicable Vercise System

	Directions for Use (DFU) for Dystonia.<br>IC4. At least 7 years old. Parent or guardian consent is required in patients who are younger than 18 years at the time of consent.
**Exclusion Criteria**	EC1. Meets any contraindication in the Vercise System locally applicable Directions for Use.
**Staged Procedures**	Subjects may undergo staged surgical procedures prior to device activation, as determined by local standards of care.
**Statistical Methods**	
**Statistical Test**	Descriptive statistics will be utilized to report on the responder rate at 26 weeks, 52 weeks, 2 years, and 3 years post first lead implant, where a responder is defined as any subject with ≥30% reduction from Baseline in BFMDRS (non-cervical dystonia) or TWSTRS (cervical dystonia) score.
**Sample Size Parameters**	The sample size for this study is calculated based on the probability of achieving a target confidence interval (CI) precision for the 30% BFMDRS/TWSTRS responder rate.<br>The study includes two subgroups based on subject diagnosis - primary dystonia and secondary dystonia. Each subgroup in the study will be analyzed separately with no pooling across subgroups. Consequently, the sample size calculation for achieving a target CI precision was performed independently for each of the two subgroups. Due to different prevalence of primary and secondary dystonia, the sample size calculation is based on the subgroup with the lower prevalence in the study population (secondary dystonia).<br>Significance level (α) 0.05<br>Assumed responder rate (π) 0.15

	Target width of 1-α confidence interval (CI)	0.30
	Probability of achieving target CI width	0.95
	Minimum subgroup prevalence	0.25
	Attrition (%)	30
	**N (both subgroups)**	**200 subjects**

3. Table of Contents

1. TITLE PAGE 1

2. PROTOCOL SYNOPSIS 2

3. TABLE OF CONTENTS 8

3.1. Table of Figures 13

3.2. Table of Tables 13

4. INTRODUCTION 14

4.1. Precedence 15

4.2. Multicenter trials and Randomized clinical studies 16

4.3. Factors affecting DBS treatment in dystonia patients 18

5. DEVICE DESCRIPTION 19

6. OBJECTIVES 21

7. ENDPOINTS 21

8. DESIGN 23

8.1. Scale and Duration 23

8.2. Treatment Assignment 24

8.3. Justification for the Study Design 24

9. SUBJECT SELECTION 24

9.1. Study Population and Eligibility 24

9.2. Inclusion Criteria 24

9.3. Exclusion Criteria 25

10. SUBJECT ACCOUNTABILITY 25

10.1. Point of Enrollment 25

10.2. Withdrawal 25

10.3. Subject Status and Classification 26

10.4. Enrollment Controls 26

11. STUDY METHODS 27

11.1. Data Collection 27

11.2. Study Candidate Screening **28**

11.3. Informed Consent **30**

11.4. Screening Assessments/Procedures (up to 90 days after Informed Consent) **30**

11.5. Baseline: Baseline Visit (up to 7 days; all subjects) & Pocket Adaptor Visit (only PA subjects) **31**

11.5.1. Baseline visit (all subjects) 31

11.5.2. Pocket Adaptor/Vercise IPG Visit 32

11.6. Implant Procedure(s) of Vercise System **32**

11.7. Device Activation **33**

11.8. Up to Week 12 (± 21 days) Visit **33**

11.9. Week 26 (± 21 days) Visit **34**

11.10. Week 52 (± 56 days) and Year 2 (± 56 days) Visit **34**

11.11. Year 3 (± 56 days) Visit: End of Study Visit **35**

11.12. Unscheduled Visits **36**

11.13. Revisions/Replacement of Leads or IPG **36**

11.14. Study Completion **36**

11.15. Source Documents **36**

12. STATISTICAL CONSIDERATIONS **37**

12.1. Analysis of Clinical Endpoints **37**

12.1.1. Responder Rate 38

12.1.2. Sample Size 38

12.2. General Statistical Methods **39**

12.2.1. Analysis Sets 39

12.2.2. Control of Systematic Error/Bias 39

12.2.3. Number of Subjects per Investigative Site 39

12.3. Data Analyses **40**

12.3.1. Interim Analyses 40

12.3.2. Subgroup Analyses 40

12.3.3. Justification of Pooling 40

12.3.4. Multivariable Analyses 40

12.3.5. Changes to Planned Analyses 41

13. DATA MANAGEMENT **41**

13.1. Data Collection, Processing, and Review **41**

13.1.1. Paper Questionnaires 41
13.1.2. Electronic Questionnaires 42
13.1.3. Direct Data Upload 42
13.2. Data Retention 42
14. STUDY ASSESSMENTS 42
14.1. Adverse Events 42
14.2. Burke-Fahn-Marsden Dystonia Rating Scale 43
14.3. Concomitant Medications 43
14.4. Clinical Global Impression of Change (CGI-C) 43
14.5. Clinical Global Impression of Change - Subject (CGI-C: Sub) 43
14.6. Clinical Global Impression of Change - Caregiver (CGI-C: Crg) 44
14.7. Global Dystonia Rating Scale (GDS) 44
14.8. Impedance Recordings (Optional) 44
14.9. Medical History 44
14.10. Montreal Cognitive Assessment (MoCA) 44
14.11. Programming Parameters 45
14.12. Resource Utilization Inventory (RUI) 45
14.13. Satisfaction with Treatment (SWT) 45
14.14. Short Form Health Survey-36 (SF-36v2), and Short Form Health Survey-10 (SF-10v2) 46
14.15. Toronto Western Spasmodic Torticollis Rating Scale (TWSTRS) 46
15. AMENDMENTS 46
16. DEVIATIONS 47
17. COMPLIANCE 47
17.1. Statement of Compliance 47
17.2. Investigator Responsibilities 47
17.2.1. Delegation of Responsibility 49
17.3. Ethics Committee 49
17.4. Sponsor Responsibilities 50
17.4.1. Role of Boston Scientific Representatives 50
17.5. Insurance 51

18. MONITORING....51
18.1. Monitoring Visits....52
18.2. Securing Compliance....53
19. POTENTIAL RISKS AND BENEFITS....53
19.1. Anticipated Adverse Device Effects....53
19.2. Contraindications....55
19.3. Anticipated Adverse Events....56
19.4. Risks Associated with the Study Device(s)....57
19.5. Risks associated with Participation in the Clinical Study....58
19.6. Possible Interactions with Concomitant Medical Treatments....58
19.7. Risk Minimization Actions....58
19.8. Anticipated Benefits....59
19.9. Risk to Benefit Rationale....59
20. SAFETY REPORTING....59
20.1. Reportable Events by investigational site to Boston Scientific....59
20.2. Definitions and Classifications....60
20.3. Relationship to Study Device(s)....63
20.4. Investigator Reporting Requirements....65
20.5. Boston Scientific Device Deficiencies....68
20.6. Reporting to Regulatory Authorities / ECs / Investigators....68
21. INFORMED CONSENT....68
22. COMMITTEES....70
22.1. Safety Monitoring Process....70
23. SUSPENSION OR TERMINATION....70
23.1. Premature Termination of the Study....70
23.2. Criteria for Premature Termination of the Study....70
23.3. Termination of Study Participation by the Investigator or Withdrawal of IRB/ EC Approval....71
23.4. Requirements for Documentation and Subject Follow-up....71
23.5. Criteria for Suspending/Terminating a Study Center....71

24. PUBLICATION POLICY **72**

25. BIBLIOGRAPHY **72**

26. ABBREVIATIONS AND DEFINITIONS **75**

26.1. Abbreviations **75**

26.2. Definitions **77**

3.1. *Table of Figures*

Figure 8.1-1: Vercise DBS Dystonia Registry Study Design 23

3.2. *Table of Tables*

Table 9-1: Inclusion Criteria 25

Table 9-2: Exclusion Criteria 25

Table 11-1: Data Collection Schedule 28

Table 11-2: Data Assessment.. 29

Table 11-3: Source Documentation Requirements 37

Table 20.2-1: Safety Definitions..60

Table 20.3-1: Criteria for Assessing Relationship of Study Device or Procedure to Adverse Event 64

Table 26-1: Abbreviations 75

Table 26-2: Definitions... 77

4. Introduction

Dystonia is a neurological condition characterized by involuntary and sustained muscle spasms which can force affected parts of the body into abnormal movements or postures. Dystonia is an umbrella term covering a broad spectrum of conditions, which can classified depending on the cause of dystonia, body areas or segments affected by dystonia, and time of onset of dystonia. Dystonia can be classified as primary or secondary depending on the cause of dystonia. Primary dystonia is idiopathic in nature whereas secondary dystonia also called as acquired dystonia results from apparent outside factors and can be attributed to a specific cause. Types of secondary dystonia include drug-induced (tardive) dystonia, dystonic cerebral palsy, dystonia resulting from Parkinson's disease (PD), metabolic disorders, or brain injury. Dystonia can be localized either to a single body region (focal), or can involve more than one body region. Depending on the continuity of body regions involved dystonia can be classified as multifocal, hemidystonia, or generalized. Types of focal dystonias' include cervical dystonia, blepharospasm, oromandibular, or laryngeal or focal hand dystonia. Multifocal dystonia involves two or more of the listed focal dystonia. In generalized dystonia, the trunk and at least 2 other sites are involved, whereas hemidystonia affects the body and limbs on one side. Dystonia can also be characterized as early-onset or late-onset depending on the time of onset of dystonia symptoms. On average, the signs and symptoms of early-onset primary dystonia appear around age 12.

The magnitude of different conditions covered under dystonia makes it challenging to determine the accurate prevalence for dystonia. Based on minimum prevalence estimates, primary dystonia is believed to be the third most frequent movement disorder after essential tremor and Parkinson's disease (Defazio et al., 2010).

In the past, for subjects who had reduced response to pharmacotherapy, thalamotomy (lesioning areas of the thalamus), and pallidotomy (lesioning areas of the globus pallidus) were the only available options for treating dystonia. However these treatment options frequently produced side effects and are now considered less advantageous due to the introduction of reversible, adjustable methods such as deep brain stimulation (DBS), and transcranial neurostimulation (TNS). In the 1990s, high-frequency deep brain stimulation was demonstrated to be safe and effective in reducing the motor complications of subjects with Parkinson's disease (Limousin, et al. 1995). In 2003, FDA approved use of DBS for the treatment of primary dystonia. Since receiving approval, the safety and efficacy of DBS for PD (Deuschl et al., 2006; Follett et al., 2010; Fraix et al., 2006; Weaver et al., 2009; Okun et al., 2012; Schuepbach et al., 2013) and dystonia (Krause et al., 2004, Markun et al. 2012; Valldeoriola et al., 2007, Krause et al., 2004; Egidi et al., 2007; Borggraefe et al., 2012, Volkmann et al., 2012) has been substantiated by numerous observational case series and clinical trials. Studies using deep brain stimulation for dystonia focus on treatment of

multitude of dystonia conditions including primary generalized (Kupsh et al., 2006; Krause et al., 2004; Heueto et al., 2010), cervical dystonia (Kiss et al., 2007), tardive dystonia (Gruber et al., 2009), and other types of secondary dystonia (Krause et al., 2004; Pretto et al., 2008; Vidailhet et al., 2009; Egidi et al., 2007; Kim et al., 2011). All these studies show highly effective results with use of DBS for treatment of dystonia.

This study will document characteristics of real-world outcomes for Boston Scientific Corporation's commercially approved Vercise System for Deep Brain Stimulation (DBS), when used according to the applicable Directions for Use. The Vercise Deep Brain Stimulation System is indicated for use in unilateral or bilateral stimulation of the subthalamic nucleus (STN) and internal globus pallidus (GPi) for treatment of primary and secondary dystonia.

4.1. *Precedence*

In 1997, the Medtronic Activa System received U.S. FDA approval for unilateral thalamic stimulation for the treatment of tremor (P960009). In 2002, it also received FDA approval (P960009/S007) for bilateral stimulation of the STN or GPi (globus pallidus interna) for the treatment of PD. Additionally, Medtronic Activa® DBS received CE Marking for the treatment of tremor in 1993, Parkinson's disease in 1998, primary dystonia in 2003, and obsessive compulsive disorder in 2009.

St. Jude Medical (ANS/St. Jude) began a U.S. study of their Libra DBS device for PD in 2005 and their results (136 subjects received bilateral stimulation of subthalamic nucleus) were published in Lancet Neurology (Okun, et al. 2012) in 2012. This device has not yet received FDA approval. St. Jude Medical received CE Marking for Parkinson's disease in 2009, and for intractable primary and secondary dystonia in April 2013.

Boston Scientific began a European study in 2010 to document subject outcomes, including effectiveness, safety, and health economic data of the Vercise™ Deep Brain Stimulation system, which is ongoing. The Vercise™ system received CE mark for treatment of Parkinson's disease in September 2012, intractable primary and secondary dystonia in September 2013 and Essential Tremor in July 2014.

4.2. *Multicenter trials and Randomized clinical studies*

Given that DBS has been commercially available for more than a decade, a large number of clinical studies have been published on this therapy. Close to a hundred[1] clinical studies have been performed in dystonia patients treated with DBS. Of these, a total of twenty-eight[2] are multi-center and randomized clinical trials (RCTs). Efficacy data from three (Valldeoriola et al., 2007; Kiss et al., 2010; Volkmann et al., 2012) recent prospective, multicenter, randomized clinical trials are discussed here. Collectively these studies present data at follow-up durations between 3 months to 5 years. At most centers, the preferred DBS target for patients with dystonia has been the globus pallidus internus (GPi). However, stimulation of the STN has also been effective for controlling dystonic symptoms. A recent study (Schjerling et al., 2013) conducted a double-blind randomized study comparing the STN and the GPi as DBS targets in the treatment of dystonia, and is also discussed here.

Valldeoriola et al., 2007 assessed the tolerability and clinical effects of bilateral pallidal DBS on motor impairment, functional disability, quality of life, pain and mood in 24 patients with medically refractory primary generalized or segmental dystonia. Bilateral GPi DBS significantly improved dystonic symptoms with both open and blind assessments. Improvement of disability, pain and mood was also observed. Seventeen of the 22 patients who completed the study achieved a good or partial response to pallidal DBS. Mood showed a mild but significant improvement after surgery and a mean 40% improvement in the physical aspects of the health related Quality of life (HRQoL). No significant changes in social function and mental health were seen. In summary, this study showed significantly improved motor symptoms, pain, quality of life and mood after GPi DBS.

Kiss et al., 2010 performed a prospective, single-blind, multi-center study assessing the efficacy and safety of bilateral GPi-DBS in 10 patients with severe, chronic, medication-resistant cervical dystonia. Two blinded neurologists assessed patients before surgery and at 6 and 12 months post-operatively using the Toronto Western Spasmodic Torticollis Rating Scale (TWSTRS). At the follow-up duration of one year, the total TWSTRS improved by 59% as compared to baseline score. There were significant improvements in quality of life (24%) and depression scores (58%), minor effects on swallowing and cognitive tests.

---

[1] As found in PubMed database (available through National Center for Biotechnology Information [NCBI] at the National Library of Medicine [NLB] located at the National Institute of Health [NIH]). Search terms: "dystonia" AND "deep brain stimulation", Limits: Clinical trial, Comparative study, Controlled clinical trial, Multicenter clinical trial, Randomized clinical trial. Date searched: 15 July, 2014

[2] As found in PubMed database (available through National Center for Biotechnology Information [NCBI] at the National Library of Medicine [NLB] located at the National Institute of Health [NIH]). Search terms: "dystonia" AND "deep brain stimulation", Limits: Multicenter clinical trial, Randomized clinical trial. Date searched: 15 July, 2014

Medications were reduced after surgery and no further botulinum toxin injections performed. Complications were mild and reversible in four patients. Some changes in neuropsychological tests were observed, although these did not impact daily life or employment. In conclusion the authors supported the efficacy and safety of GPi-DBS for the treatment of patients with severe and prolonged cervical dystonia who have failed medical management.

Volkmann et al., 2012 assessed safety and efficacy of GPi stimulation in patients with primary generalized or segmental dystonia prospectively followed up for 5 years in a controlled multicenter trial. Forty (40) patients were randomly assigned to either sham neurostimulation or neurostimulation of the internal globus pallidus for a period of 3 months and thereafter all patients completed 6 months of active neurostimulation. The primary endpoint was the change in dystonia severity at 3 years and 5 years as assessed by open-label ratings of the Burke-Fahn-Marsden dystonia rating scale (BFMDRS) motor score compared with the preoperative baseline and the 6-month visit. The primary endpoint was analyzed on an intent-to-treat basis. An intent-to-treat analysis including all patients from the parent trial showed significant improvements in dystonia severity at 3 years and 5 years compared with baseline, which corresponded 47·9% improvement at 6 months (n=40); 61·1% improvement at 3 years (n=31); 57·8% improvement at 5 years (n=32). 49 new adverse events occurred between 6 months and 5 years. Authors concluded sustained benefits and an acceptable safety profile for the treated dystonia population.

Schjerling et al., 2013 compared the subthalamic nucleus (STN) with the globus pallidus internus (GPi) as a stimulation target for deep brain stimulation (DBS) for medically refractory dystonia. In a prospective double-blind crossover study, electrodes were bilaterally implanted in the STN and GPi of 12 patients with focal, multifocal, or generalized dystonia. Each patient was randomly selected to undergo initial bilateral stimulation of either the STN or the GPi for 6 months, followed by bilateral stimulation of the other nucleus for another 6 months. Preoperative and postoperative ratings were assessed by using the Burke-Fahn-Marsden Dystonia Rating Scale (BFMDRS) and video recordings. Quality of life was evaluated by using questionnaires (36-item Short Form Health Survey). Supplemental Toronto Western Spasmodic Torticollis Rating Scale (TWSTRS) scores were assessed for patients with focal dystonia (torticollis) by examining the video recordings. On average for all patients, DBS significantly improved the BFMDRS movement scores ($p < 0.05$) and quality of life physical scores ($p < 0.01$). At 6 months, BFMDRS movement score with STN simulation had a non-significant improvement of the TWSTRS movement score as compared to GPi simulation. Quality of life did not differ significantly regardless of which nucleus was stimulated. The authors concluded that further studies are necessary before the optimal target can be concluded.

4.3. *Factors affecting DBS treatment in dystonia patients*

Improvement in DBS treatment for dystonia is variable from one patient to another, and could depend on factors related to the disease state or treatment. A number of studies have focused on understanding factors that could possibly have predictive value on the magnitude of improvement with DBS treatment for dystonia. These factors include dystonia etiology, and duration of disease. A recent study also showed higher volume of activation of GPi in highly improved patients compared to less improved patients.

As mentioned earlier, primary dystonia is idiopathic in nature, whereas secondary dystonia results from apparent outside factors and can be attributed to a specific cause. A group of early-onset primary dystonia syndromes can be classified based on clinical presentation and assigned to a DYT number. At present about 20 monogenetic dystonia syndromes, include seven primary dystonia syndromes (DYT1, 2, 4, 6, 7, 13, and 17) have been classified. Of these dystonia syndromes, the genes TOR1A (causing DYT1), and THAP (DYT6) have been identified. Primary dystonia patients, specifically with DYT1 positive mutation have been typically shown to have better outcomes as compared to secondary dystonia patients (Borggraefe et al., 2012, Egidi et al., 2007, Krause et al., 2004, Markun et al. 2007, Lumsden et al, 2013). Treatment outcome in secondary dystonia patients has been quite poor (Krause et al., 2004; Pretto et al., 2008; Vidailhet et al., 2009), with the exception of drug-induced secondary dystonia patients who seem to benefit significantly from DBS treatment (Egidi et al., 2007). Considering the variability in treatment effect, a subgroup analysis between primary and secondary dystonia patients will be performed. Further exploratory analysis in other primary monogenetic dystonia syndrome population is also intended.

A number of studies show that duration of disease in primary dystonia correlates with magnitude of improvement. Patients with shorter duration of the disease may benefit more than patients who have exhibited symptoms for a longer duration (Egidi et al., 2007; Markun,et al., 2007; Andrews et al., 2010; Isaias et al., 2011; Lumsden et al, 2013). Younger patients with short disease duration benefit more and faster than older patients (Isaias et al., 2011) Lumsden et al., 2013 recommends DBS implantation in early, preferably within 5 years of onset, are made to maximize benefits and reduce the childhood experience of dystonia. Based on this evidence, analysis on the effect of disease duration on DBS treatment will be performed.

In summary, the results from these recent multi-center, randomized controlled trials provide solid evidence in support of the effectiveness and safety of DBS as a therapy for PD. Further, studies analyzing the effect of the disease factors on treatment effect provide the basis for

need for subgroup analysis based on the disease factors of this population. This review is used as the basis for the design of this study.

5. Device Description

The Boston Scientific Vercise™ DBS System received CE mark approval in September 2012 for treatment of Parkinson’s disease, in September 2013 for treatment of intractable primary and secondary dystonia and in July 2014 for Essential Tremor.

The implantable portion of the Vercise™ System includes leads for bilateral stimulation and extensions that allow the leads to be extended to reach the Implantable Pulse Generator (IPG) near the clavicle. The electrode array is typically placed into the brain, targeting the subthalamic nucleus (STN) or Globus Pallidus (GPi). Leads can provide stimulation in a ring mode (around the lead) or if the directional lead is used in a directional mode (away from the lead). Once the leads are placed, they are typically secured using physician’s standard lead fixation technique. A SureTek™ Burr Hole Cover may be used. Since the leads are often placed under the scalp for some time before being connected to lead extensions or IPG, lead boots will be provided to cover the proximal ends of the leads during this period.

Surgical tools for the Vercise™ System include a tunneler (similar to the commercially available Precision SCS tunneling tool). A lead stop (which may be attached to the lead to allow the lead to be located at the appropriate depth in the tissue during its initial placement) will also be provided.

External devices including a Clinician Programmer, Remote Control and, if needed, a charging system will also be provided for patient/physician use. A charging collar has been designed to facilitate the charging of the IPG in the sub-clavicular location.

The Pocket Adaptor allows the Vercise IPG to be used in subjects implanted with non-BSC leads.

6. Objectives

To compile characteristics of real-world outcomes, economic value and technical performance of Boston Scientific Corporation's commercially approved Vercise^{TM} DBS system, when used according to applicable Directions for Use (DFU)

7. Endpoints

Clinical Endpoints

All clinical endpoints will be analyzed separately for subjects with primary and secondary dystonia sub-groups. The Statistical Analysis Plan will provide details on whether descriptive statistics or statistical hypothesis testing will be performed for each endpoint. The specific assessment or questionnaire to be used depends on the

subjects' dystonia classification and age as detailed in Table 11-2. The following clinical endpoints will be analyzed for each sub-group of dystonia separately (Primary vs. Secondary):

- Proportion of subjects with 30% or greater reduction in Baseline BFMDRS or TWSTRS score at 26 weeks, 52 weeks, 2 years, and 3 years post first lead placement
- Change in BFMDRS scores from Baseline to 26 weeks, 52 weeks, 2 years, and 3 years post first lead placement
- Change in TWSTRS scores from Baseline to 26 weeks, 52 weeks, 2 years, and 3 years post first lead placement
- Change in SF-36v2 score (SF-10v2 in patients under the age of 18 years at the time of consent) from Baseline to 26 weeks, 52 weeks, 2 years, and 3 years post first lead placement
- Change in GDS scores from Baseline to 26 weeks, 52 weeks, 2 years, and 3 years post first lead placement.
- Clinical Global Impression of Change (CGI-C) rating score, as assessed by neurologist, at 26 weeks, 52 weeks, 2 years, and 3 years post first lead placement
- Clinical Global Impression of Change – Subject (CGI-C: Sub), as assessed by subject, at 26 weeks, 52 weeks, 2 years, and 3 years post first lead placement
- Clinical Global Impression of Change – Caregiver (CGI-C: Crg), as assessed by caregiver, at 26 weeks, 52 weeks, 2 years, and 3 years post first lead placement

Safety Parameters

Rates of occurrence of all serious adverse events (SAEs) and all adverse device effects (ADEs), including serious adverse device effects (SADEs) and unanticipated serious adverse device effects (USADEs) at 3 years post first lead placement

Health Economics Endpoints

- Total cost of treatment and resource utilization from Baseline through end of study (RUI)
- Change in economic value from Baseline to 26 weeks, 52 weeks, 2 years and 3 years post first lead placement.

8. Design

The Vercise DBS Registry for Dystonia is a prospective, on-label, multi-center, international registry.

8.1. *Scale and Duration*

The study will be conducted at up to 40 international centers. Up to 200 subjects will be implanted. Follow-up of each subject will continue for three years after first lead placement. The study may require up to five calendar years to complete.

Figure 8.1-1: Vercise DBS Dystonia Registry Study Design

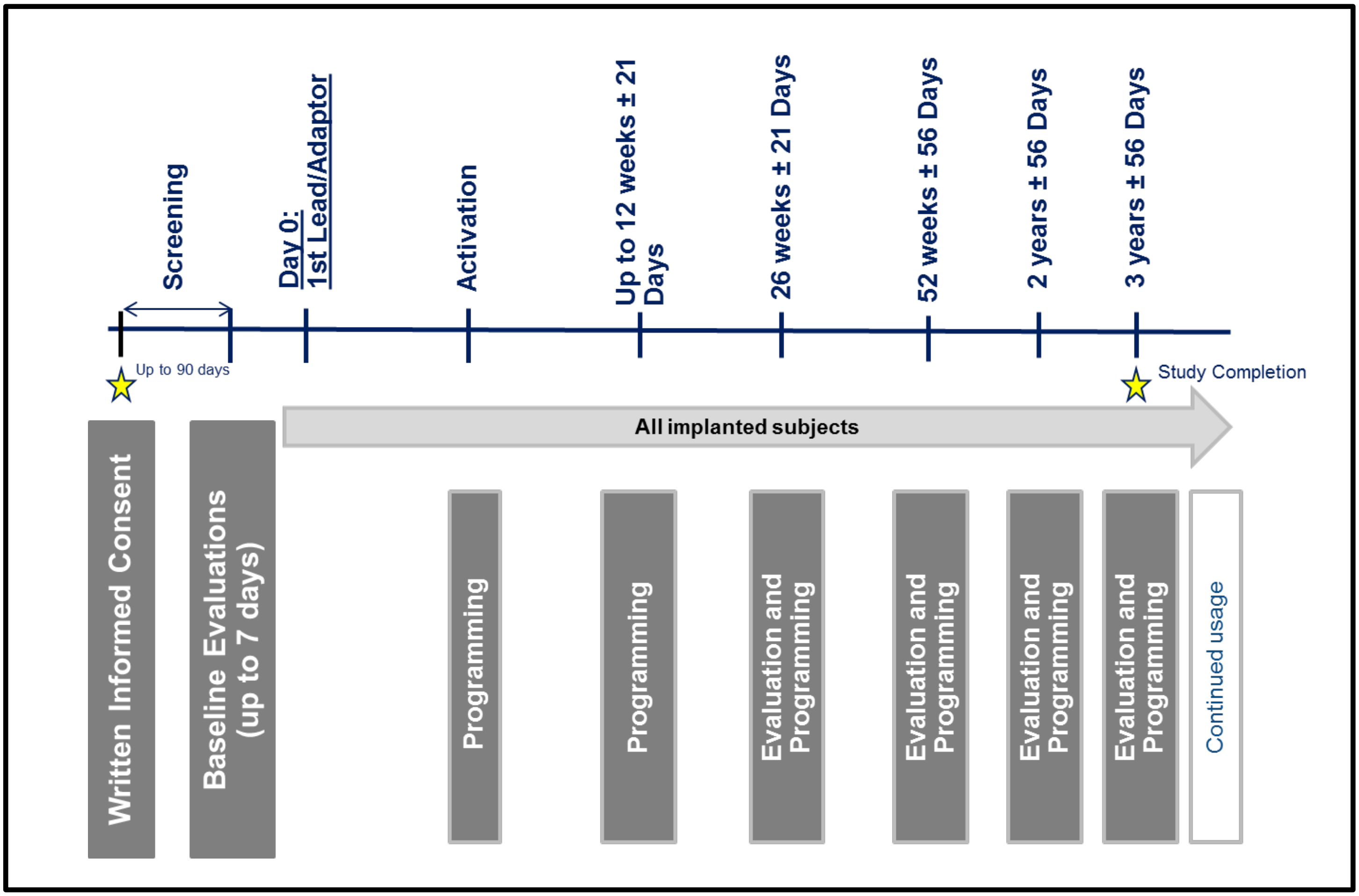

8.2. *Treatment Assignment*

Subjects who provide written informed consent will be considered enrolled in the study and therefore assigned a unique subject identifier in the Electronic Data Capture (EDC) system.

Consented subjects who successfully pass all screening requirements will receive the Vercise^{TM} System.

8.3. *Justification for the Study Design*

All devices used in this registry are approved for commercial use.

The endpoints of this registry are intended to provide a broad evidence base to assess long-term clinical and economic outcomes of the Vercise System in a large number of subjects representing 'real-world' use patterns. The registry will also bridge the existing evidence gap in disease management, treatment effectiveness, health economic value, and patient outcomes for on-label use of the system.

9. Subject Selection

9.1. *Study Population and Eligibility*

Up to 200 implanted subjects may be included in the registry.

Subjects will generally be recruited from physician's practice and will be eligible to receive Deep Brain Stimulation to treat their Dystonia using the commercially-approved BSC Vercise System per local DFU.

9.2. *Inclusion Criteria*

Subjects who meet all of the following criteria (see Table 9-1) may be given consideration for inclusion in this clinical investigation, provided no exclusion criterion (see Section 9.3) is met.

Table 9-1: Inclusion Criteria

**Inclusion Criteria**	IC1. Understands the study requirements and the treatment procedures and provides written informed consent before any study-specific tests or procedures are performed.<br>IC2. Receive currently available Vercise System and any new CE marked components (i.e., leads, extension, Pocket Adaptor, CP, etc.) compatible with the Vercise System<br>IC3. Meets criteria established in the locally applicable Vercise System Directions for Use (DFU) for Dystonia.<br>IC4. At least 7 years old. Parent or guardian consent is required in patients who are younger than 18 years at the time of consent.

9.3. *Exclusion Criteria*

Subjects who meet the following criterion (Table 9-2) will be excluded from this clinical study.

Table 9-2: Exclusion Criteria

**Exclusion Criteria**	EC1. Meets any contraindication in the Vercise System locally applicable Directions for Use.

10. Subject Accountability

10.1. *Point of Enrollment*

A patient will be considered enrolled in this study after he/she signs and dates the informed consent form (ICF). No study-related procedures or assessments can take place until the informed consent form is signed.

10.2. *Withdrawal*

All subjects enrolled in the clinical study (including those withdrawn from the clinical study or lost to follow-up) shall be accounted for and documented. If a subject withdraws from the clinical investigation, the reason(s) shall be reported. If such withdrawal is due to problems

related to investigational device safety or performance, the investigator shall use local treatment protocols to determine the subject's options for therapy.

Reasons for withdrawal could include but are not limited to

- physician discretion,
- subject choice to withdraw consent; in case of subjects under the age of 18 years old, parent/guardian choice to withdraw consent will be considered a valid reason for withdrawal.
- subject's failure to meet registry inclusion or exclusion criteria after enrollment but prior to system implant,
- failure to receive a BSC DBS system,
- lost to follow-up, or
- death.

While study withdrawal is discouraged, subjects may withdraw from the study at any time, with or without reason, and without prejudice to further treatment.

All applicable case report forms (CRFs) up to the point of subject withdrawal and an "End of Study" form must be completed. Any subject deemed "lost to follow-up" should have a minimum of three documented attempts to contact him/her prior to completion of the "End of Study" form.

Additional data may no longer be collected after the point at which a subject has been withdrawn from the study or withdraws consent, for whatever reason. All open adverse events should be closed or documented as unresolved. Data collected up to the point of subject withdrawal may be used and analyzed.

Subjects withdrawn after completing the implant procedure will not be replaced and will be included in the site's overall total for implanted subjects.

10.3. *Subject Status and Classification*

A subject is considered enrolled after a signed informed consent form (ICF) has been obtained. Study participation will end when an implanted subjects completes their 3 Year follow up visit.

10.4. *Enrollment Controls*

Enrollment will remain open until one of the following events occurs:

- 200 subjects are implanted.

- The study is terminated at any time, at the Sponsor's discretion.

Enrollment controls will be implemented per the Enrollment Communication Plan developed for this study.

11. Study Methods

11.1. *Data Collection*

The data collection schedule is shown in Table 11-1. Assessments and questionnaires should be utilized based on subject classification and age, and as highlighted in Table 11-2.

Specific questionnaires or parts of questionnaires may not be applicable due to age or type of dystonia.

Table 11-1: Data Collection Schedule

Data collection variable/ Study assessment*	Screening	Pocket Adaptor (PA) with MDT IPG ON	Baseline visit (Up to 90 days post ICF)	Implant procedures Day 0 (Leads, IPG)	Activation	Week 12 Visit (84 days ± 21)	Week 26 Visit (182 days ± 21)	Week 52 Visit (365 days ± 56)	Year 2 Visit (730 days ± 56)	Year 3 Visit (1095 days ± 56)	Unscheduled visits (anytime)
Adverse Event (AE) †		X	X	X	X	X	X	X	X	X	X
Burke-Fahn-Marsden Dystonia Rating Scale (BFMDRS)		X	X				X	X	X	X	
Clinical Global Impression of Change (CGI-C)‡		X					X	X	X	X	
Clinical Global Impression of Change (CGI-C: Sub)‡		X					X	X	X	X	
Clinical Global Impression of Change (CGI-C: Crg)‡		O					O	O	O	O	
Concomitant Medications			X		X		X	X	X	X	
Demographics			X								
Global Dystonia Rating Scale (GDS)		X	X				X	X	X	X	
Genetic testing results	O										
Inclusion/exclusion criteria evaluation	X										
Informed consent form§	X										
Medical history			X								
Monopolar review					O						
Montreal Cognitive Assessment (MoCA)		X	X				X	X	X	X	
Procedural information				X							
Programming parameters		X			X		X	X	X	X	
Impedance recordings		O			O		O	O	O	O	
Resource Utilization Inventory (RUI)		X	X[#]				X	X	X	X	
Satisfaction with Treatment (SWT)‡		X					X	X	X	X	
Short Form Health Survey-10 (SF-10v2)		X	X[#]				X	X	X	X	
Short Form Health Survey-36 (SF-36v2)		X	X[#]				X	X	X	X	
Toronto Western Spasmodic Torticollis Rating Scale (TWSTRS)		X	X				X	X	X	X	

* Assessments and questionnaires should be utilized based on subject classification and age, and as highlighted in Table 11-2. Baseline Clinician administered assessments done as part of standard of care screening to determine eligibility for DBS up to 180 days prior to baseline may be used for this Baseline Visit (no repetition needed).

Pocket Adaptor (PA): Subjects receiving a pocket adaptor to connect non-BSC leads to BSC IPG

X = required; O = optional

† Only adverse events related to the device, stimulation, or procedure, and serious adverse events will be recorded

‡ In PA patients, data for CGI-C, and SWT at Pocket Adaptor Visit will be collected in comparison with patient condition before receiving non-BSC implant. At follow-up visits, these data will be collected in comparison to patient STIM OFF condition at Baseline.

[#] For PA patients, not to be completed at Baseline

§ In subjects younger than 18 years of age, form should be completed by both the subject and the parent/guardian.

Table 11-2: Data Assessment

Assessment and Questionnaires	Subject classification by diagnoses and age
Burke-Fahn-Marsden Dystonia Rating Scale (BFMDRS)	Subjects with non-cervical dystonia;
Clinical Global Impression of Change (Clinician - CGI-C)	All subjects
Clinical Global Impression of Change (Subject - CGI-C: Sub)	All subjects
Clinical Global Impression of Change (Caregiver – CGI-C:Crg) OPTIONAL	All subjects
Global Dystonia Rating Scale (GDS)	All subjects
Montreal Cognitive Assessment (MoCA)	All subjects
Resource Utilization Inventory (RUI)	All subjects
Short Form Health Survey-36 (SF-36v2)	Subjects aged 18 years and older at time of consent
Short Form Health Survey-10 (SF-10v2)	Subjects under the age of 18 years at time of consent
Satisfaction with Treatment (SWT)	All subjects
Toronto Western Spasmodic Torticollis Rating Scale (TWSTRS)	Subjects with cervical dystonia; 18 years and older at time of consent

11.2. *Study Candidate Screening*

All interested subjects will undergo screening during which their eligibility for the study will be determined.

11.3. *Informed Consent*

Written informed consent must be obtained from all potential study candidates. Parent or guardian consent is required in patients who are between 7- 18 years old at the time of consent. A subject is considered enrolled only after the subject signs and dates the Informed Consent Form (ICF).

- Subjects and/or subjects' parent/guardian will be asked to sign the Informed Consent form before any study-specific tests or procedures are performed;
- The context of the study must be fully explained and the subjects and/or subjects' parent/guardian must be given the opportunity to ask questions and have those questions answered to their satisfaction;
- Study personnel should explain that even if a subject agrees and/or subjects' parent/guardian agrees to participate in the study and signs an ICF, certain screening procedures might demonstrate that the subject is not eligible to continue participation;
- The Informed Consent form is study-specific and must be approved by the Independent Ethics Committee (IEC) and the sponsor.
- Written informed consent must be recorded appropriately by means of the subject's and/or subjects' parent/guardian dated signature.

11.4. *Screening Assessments/Procedures (up to 90 days after Informed Consent)*

Enrolled subjects should undergo screening to determine eligibility for participation in the study. Subjects who meet all inclusion criteria (as described in Section 9.2) may be given consideration for participation, provided no exclusion criterion (as described in Section 9.3) is met.

The Written Informed Consent form, screening assessments/procedures, and the Baseline Visit may all be completed on the same day.

Candidate screening assessments/ and or procedures should not take any longer than 90 days from the start of the screening period.

11.5. *Baseline: Baseline Visit (up to 7 days; all subjects) & Pocket Adaptor Visit (only PA subjects)*

During the Baseline Visit, a detailed evaluation of subjects' disease state and its impact on their health will be performed. Evaluation of the subjects disease state includes assessment of the type of dystonia (primary, secondary, or idiopathic), and specific diagnoses (e.g. cervical dystonia, blepharospasm, generalized dystonia, focal dystonia etc.) per standard of care at the investigating site. Subjects or the subjects' parent/guardian (for subjects younger than 18 years) will be asked to complete certain evaluations related to their dystonia symptoms, quality of life, mental health status, etc. Subjects' medications for the treatment of dystonia will be documented. It is recommended that genetic testing for DYT status be performed per standard of care.

The Written Informed Consent form may be obtained during the Baseline Visit provided that all screening assessments/procedures are also done on the same day. All baseline evaluations/ assessments/ tests must be completed within 7 days of the patient's first baseline visit. Baseline assessments done as part of standard of care screening to determine eligibility for DBS up to 90 days prior to consent may be used for this Baseline Visit (no repetition needed).

11.5.1. **Baseline visit (all subjects)**

The following general assessments will be completed during the Baseline Visit. Subjects receiving the Pocket Adaptor will be asked to turn OFF stimulation to obtain their Baseline Visit data. It may take up to 7 days of washout, per investigator discretion, before subjects reached their baseline status with stimulation OFF. The specific assessment or questionnaire delivered to the subject depends on subject classification and age, and guidance regarding these is provided in Table 11-2.

- Medical History
- Concomitant Medications
- Demographic information – Gender, age at consent, level of education, and work/school status.
- Burke-Fahn-Marsden Dystonia Rating Scale (BFMDRS)
- Global Dystonia Rating Scale (GDS)
- Montreal Cognitive Assessment (MoCA)
- Resource Utilization Inventory (RUI) (only for patients without Pocket Adaptor)

- Short Form Health Survey-36 (SF-36 v2) or Short Form Health Survey-10 (SF-10v2) (only for patients without Pocket Adaptor)
- Toronto Western Spasmodic Torticollis Rating Scale (TWSTRS)

Subjects will also be asked about any adverse events since their last study visit (if any).

11.5.2. **Pocket Adaptor/Vercise IPG Visit**

Subjects who are going to receive Vercise IPG as a replacement to a non-Boston Scientific IPG, and that may connect the Vercise IPG to components not manufactured by Boston Scientific will have an additional visit at the time of Baseline:

The following assessments, depending on Dystonia type, will be completed with their existing non-BSC system stimulation ON based on status of their non-BSC IPG and subject approval. These assessments may be completed during the Screening Period.

- Burke-Fahn-Marsden Dystonia Rating Scale (BFMDRS)
- Clinical Global Impression of Change (CGI-C)
- Clinical Global Impression of Change – Subject (CGI-C: Sub)
- Clinical Global Impression of Change – Caregiver (CGI-C: Crg) optional
- Global Dystonia Rating Scale (GDS)
- Montreal Cognitive Assessment (MoCA)
- Resource Utilization Inventory (RUI)
- Satisfaction with Treatment Short Form Health Survey-36 (SF-36 v2) or Short Form Health Survey-10 (SF-10v2)
- Toronto Western Spasmodic Torticollis Rating Scale (TWSTRS)

Following completion of assessments, subjects will be scheduled for a Vercise implant.

11.6. *Implant Procedure(s) of Vercise System*

Detailed directions for use will be available to each investigator and investigational site in the form of instructional manuals and training by BSC staff. Because DBS is a well-established therapy, neurosurgeons, neurologists, and their staffs have developed their own best procedures and practices for implant, activation, and programming. Standard practices, per local DFUs will be followed for the placement of the Vercise system.

The date of the first lead placement will be considered as Day 0. Alternatively, for subjects receiving BSC IPG and BSC Pocket adaptor as a replacement to non-BSC IPG, the day of BSC IPG implant will be considered as Day 0. Also, if newly marked CE active components- such as the lead or IPG are used, then that day shall be considered as Day 0. All subsequent study schedule visits will occur based on the timing of the Day 0 visit.

Standard practices, per local DFUs will be followed for the placement of the Vercise system.

Post-operative imaging is recommended to be done within 3 months following placement of leads to confirm lead position. A computerized tomography (CT scan) with at least 1.25 mm slice thickness is recommended.

The implant procedure must be performed within 6 months of the baseline.

11.7. *Device Activation*

The Vercise system will be activated following completion of the system implant.

Subjects will be asked about any adverse events since their last study visit. Any changes to subjects' medications will be documented.

The device stimulation parameters will be chosen based on subject and physician preference per local site standards/DFU. Subjects or subjects' parent/ guardian will be instructed on the use of the remote control and charging system. Any precautions/restrictions will be discussed.

A monopolar review (optional) capturing efficacy and side effect thresholds will be conducted at this visit to determine initial settings (optional). The final programming parameters (for example, rate, pulse width, etc.) will be documented. Impedance measurements will also be performed and recorded.

Subjects may have as many clinic visits as required for optimization of programming.

11.8. *Up to Week 12 (± 21 days) Visit*

Several visits following activation of the Vercise system may be made for optimization of therapy and/or management of adverse events. These visits will be scheduled with the neurologist (or neurosurgeon as needed).

Subjects will be asked about any adverse events since their last study visit.

The subjects' device may be checked for performance including an impedance check and programming as needed. The programming parameters and impedances will be documented if required per the local site's standard of care.

11.9. *Week 26 (± 21 days) Visit*

This visit will be scheduled with the neurologist.

Subjects will be asked about any adverse events since their last study visit. Any changes to subjects' medications will be documented.

The following general assessments must be completed. The specific assessment or questionnaire delivered to the subject depends on subject diagnoses, and guidance regarding these is provided in Table 11-2.

- Burke-Fahn-Marsden Dystonia Rating Scale (BFMDRS)
- Clinical Global Impression of Change (CGI-C)
- Clinical Global Impression of Change – Subject (CGI-C: Sub)
- Clinical Global Impression of Change – Caregiver (CGI-C: Crg) optional
- Global Dystonia Rating Scale (GDS)
- Montreal Cognitive Assessment (MoCA)
- Resource Utilization Inventory (RUI)
- Satisfaction with Treatment (SWT)
- Short Form Health Survey-36 (SF-36 v2) or Short Form Health Survey-10 (SF-10v2)
- Toronto Western Spasmodic Torticollis Rating Scale (TWSTRS)

The subjects' device may be checked for performance including an impedance check and programming as needed. The programming parameters and impedances will be documented. if required per the local site's standard of care.

11.10. *Week 52 (± 56 days) and Year 2 (± 56 days) Visit*

The Week 52 (Year 1) and Year 2 visits will be scheduled with the neurologist.

Subjects will be asked about any adverse events since their last study visit. The following general assessments must be completed. The specific assessment or questionnaire delivered to the subject depends on subject diagnoses, and guidance regarding these is provided in Table 11-2.

- Burke-Fahn-Marsden Dystonia Rating Scale (BFMDRS)
- Clinical Global Impression of Change (CGI-C)
- Clinical Global Impression of Change – Subject (CGI-C: Sub)
- Clinical Global Impression of Change – Caregiver (CGI-C: Crg) optional

- Global Dystonia Rating Scale (GDS)
- Montreal Cognitive Assessment (MoCA)
- Resource Utilization Inventory (RUI)
- Satisfaction with Treatment (SWT)
- Short Form Health Survey-36 (SF-36 v2) or Short Form Health Survey-10 (SF-10v2)
- Toronto Western Spasmodic Torticollis Rating Scale (TWSTRS)

The subjects' device may be checked for performance including an impedance check and programming as needed. The programming parameters and impedances will be documented if required per the local site's standard of care.

11.11. *Year 3 (± 56 days) Visit: End of Study Visit*

This visit will be scheduled with the neurologist. Year 3 Visit will be the last study visit.

Subjects will be asked about any adverse events since their last study visit. If anti-dystonic medications were collected during the baseline visit, then any changes to these will be documented. The following general assessments must be completed. The specific assessment or questionnaire delivered to the subject depends on subject diagnoses, and guidance regarding these is provided in Table 11-2.

- Burke-Fahn-Marsden Dystonia Rating Scale (BFMDRS)
- Clinical Global Impression of Change (CGI-C)
- Clinical Global Impression of Change – Subject (CGI-C: Sub)
- Clinical Global Impression of Change – Caregiver (CGI-C: Crg) optional
- Global Dystonia Rating Scale (GDS)
- Montreal Cognitive Assessment (MoCA)
- Resource Utilization Inventory (RUI)
- Satisfaction with Treatment (SWT)
- Short Form Health Survey-36 (SF-36 v2) or Short Form Health Survey-10 (SF-10v2)
- Toronto Western Spasmodic Torticollis Rating Scale (TWSTRS)

The subjects' device may be checked for performance including an impedance check and programming, as needed. The programming parameters and impedances will be documented if required per the local site's standard of care.

11.12. *Unscheduled Visits*

Unscheduled visits may be made at any time for reasons including reprogramming or for evaluation of a possible adverse event (AE). Each unscheduled visit should be documented with the reason for the visit stated. Details of adverse event must be recorded. If subjects' device was tested or programmed, the final programming parameters and impedances will be documented.

11.13. *Revisions/Replacement of Leads or IPG*

It is possible that leads may be placed incorrectly, migrate, fail or get infected. This may require repositioning, replacement or explant. It is also possible that the IPG may fail and require replacement or explant, or need repositioning due to migration, discomfort, etc. Any replacements, revisions or explants performed during the course of the study should be documented.

11.14. *Study Completion*

De-novo subjects will be followed for 3 years after first lead implant, and subjects with Pocket Adaptor will be followed for 3 years post Vercise IPG placement. The study will be considered complete once all subjects have completed Year 3 Visit.

11.15. *Source Documents*

Where copies of the original source document as well as printouts of original electronic source documents are retained, these shall be signed and dated by a member of the investigation center team with a statement that it is a true reproduction of the original source document.

All source documentation (Table 11-3) will be retained at the investigational site. The type of the source documentation will be specific for each study visit and whether the visit was conducted at the neurologist's or a neurosurgeon's office. When neurologist and neurosurgeons are not at the same institution, each office will be responsible for keeping source documentation related to only those study visits conducted at their institution. Examples of source documents include, but are not limited to the following:

- Informed consent form and consent process documentation;
- Documentation of medical history components;
- Medical records from clinical visits (i.e. progress notes, etc.) and operative procedures (i.e. Operative Report, etc.);
- Study-specific source document worksheets (if applicable);

- Laboratory/Test Results, if applicable;
- Neuropsychological and psychiatric tests results;
- Questionnaires such as SF-36v2, etc.;
- Programming information from Clinician Programmer following programming/testing.
- Imaging (i.e. pre-procedure CT scan and/or MRI, post-OP CT scan of lead position).

Table 11-3: Source Documentation Requirements

**Requirement**	**Disposition**
Informed Consent Form	**Retained at investigational site**
Consent Process Documentation	**Retained at investigational site**
Inclusion & Exclusion Criteria Documentation	**Retained at investigational site**
Medical Records	**Retained at investigational site**
Patient Questionnaires	**Retained at Investigational site**
Laboratory/Test Results (if applicable)	**Retained at investigational site**
Imaging films/prints (if applicable)	**Retained at investigational site**
Technical Source Form	**Retained at investigational site**
Clinician Questionnaires	**Retained at investigational site**

12. Statistical Considerations

12.1. *Analysis of Clinical Endpoints*

A number of clinical endpoints are included in this study, as described in Section 7. Subjects in this study will be diagnosed for primary or secondary dystonia, and will be assigned to either of the two subgroups. All clinical endpoints will be evaluated separately for these two subgroups, and will be summarized using descriptive statistics for continuous variables (e.g., mean, standard deviation, N, minimum, maximum) and frequency tables or proportions for discrete variables. Estimates of all endpoints will be reported, as well as the 95% confidence intervals.

12.1.1. **Responder Rate**

A responder is defined as a subject with 30% or greater reduction in Baseline BFMDRS or TWSTRS score in non-cervical and cervical dystonia subjects, respectively. The responder rate is calculated as the number of responders divided by the number of subjects within each subgroup. Because the number of responders follows a binomial distribution, the 95% confidence interval for the proportion of responders will be calculated using the Clopper-Pearson method.

12.1.2. **Sample Size**

The sample size is calculated as the number of subjects needed to achieve a specific target precision for the responder rate. The goal is to obtain an estimate of the responder rate while limiting the uncertainty surrounding that estimate. The precision of the responder rate is measured by the width of its 95% confidence interval, with larger widths indicating lack of precision. The study is designed such that the difference between the upper and lower 95% confidence bounds for the responder rate is, with 95% certainty, less than the pre-specified target. That is, given the sample size parameters; there is a 95% chance that the target precision for the primary endpoint will be achieved.

Due to different prevalence of primary and secondary dystonia, the sample size calculation is based on the subgroup with the lower prevalence in the study population. This is the secondary dystonia group.

Parameters for the sample size calculations (where N is the total number of implanted subjects) are shown in Table 12.1.

Table 12.1: Sample Size Parameters

Significance level (α)	0.05
Assumed responder rate (π)	0.15
Target width of 1-α confidence interval (CI)	0.30
Probability of achieving target CI width	0.95
Subgroup prevalence	0.25
Attrition (%)	30
**Total N (both subgroups)**	**200 Subjects**

12.2. *General Statistical Methods*

12.2.1. **Analysis Sets**

All clinical endpoints will be analyzed on both intent-to-treat and a per-protocol basis. Safety endpoints will be analyzed in the safety analysis set.

- Intent-to-Treat (ITT)

  In the intent-to-treat analysis, all enrolled subjects who undergo a Vercise DBS implant procedure will be included in the analysis.

- Per-Protocol

  In the per-protocol analysis, only subjects completing all scheduled protocol visits through 52 weeks with no major protocol deviations will be included in the analysis. A comprehensive list of major protocol deviations will be provided in the Statistical Analysis Plan.

- Safety Analysis Set

  In the safety analysis, all subjects who sign the IRB/EC-approved written Informed Consent form will be included.

12.2.2. **Control of Systematic Error/Bias**

Selection of patients will be made from the Investigator's usual patient population, including referred patients. All patients meeting the inclusion/exclusion criteria and having signed the Informed Consent Form will be eligible for participation in the study. The reasons for exclusion, for patients who sign an informed consent form, but are not enrolled, will be indicated in the patient screening log. Consequently, consecutively eligible patients will be enrolled in the study, minimizing selection bias. BSC will report to the relevant competent authorities any evidence of fraud, including deliberate tampering with the selection of patients.

12.2.3. **Number of Subjects per Investigative Site**

Enrollment will be competitive across sites, with an initial maximum of 10 implanted subjects at any one site. The per-site implant cap of 10 subjects may be increased upon Sponsor approval. At the time when the study-wide enrollment cap is reached, further enrollment into the study will cease regardless of whether individual sites have reached their per-site cap.

12.3. *Data Analyses*

Post-procedure information will be collected at regularly scheduled follow-up examinations as detailed in the clinical trial schedule and will be summarized using descriptive statistics for continuous variables (e.g., mean, standard deviation, n, minimum, maximum) and frequency tables or proportions for discrete variables.

All statistical analyses will be done using the SAS System software, version 8.1 or later (Copyright © 2000 SAS Institute Inc., SAS Campus Drive, Cary, North Carolina 27513, USA. All rights reserved).

12.3.1. **Interim Analyses**

The two subgroups in the study (primary dystonia and secondary dystonia) will be tested separately and no pooling across subgroups will be performed. Consequently, analysis will proceed when sufficient power is reached separately for each subgroup.

12.3.2. **Subgroup Analyses**

Results will be summarized for the following subgroups of subjects. These subgroup analyses are not powered, and will be for exploratory purposes only. These analyses are subject to availability of data.

- Age at surgery
- Age of disease onset
- Duration of disease
- Dystonia Classification
- Genetic status
- STN versus GPi implanted subjects

12.3.3. **Justification of Pooling**

Analyses will be performed using data pooled across institutions. Multivariate analysis techniques, including contingency tables and logistic regression for binary outcomes and analysis of variance for continuous measures, will be used to assess differences among study institutions to justify pooling data across institutions.

12.3.4. **Multivariable Analyses**

Multivariate models will be used to assess the predictability of outcome from the risk factors. Details will be provided in the Statistical Analysis Plan.

12.3.5. Changes to Planned Analyses

Any changes to the planned statistical analyses made prior to performing the analysis will be documented in an amended Statistical Analysis Plan approved prior to performing the analysis. Changes from the planned statistical methods after performing the analysis will be documented in the clinical study report along with a reason for the deviation.

13. Data Management

13.1. *Data Collection, Processing, and Review*

Subject data will be recorded in a limited access secure electronic data capture (EDC) system.

The clinical database will reside on a production server hosted by Medidata Solutions Inc. The associated Medidata/RAVE software and database have been designed to meet regulatory compliance for deployment as part of a validated system compliant with laws and regulations applicable to the conduct of clinical studies pertaining to the use of electronic records and signatures. All changes made to the clinical data will be captured in an electronic audit trial and available for review by Boston Scientific Corporation (BSC) or its representative. The Investigator provides his/her electronic signature on the appropriate electronic case report forms (eCRFs) in compliance with local regulations. If required by local regulations, a written signature on printouts of the eCRFs is also performed. Changes to data previously submitted to the sponsor require a new signature by the Investigator acknowledging and approving the changes.

Visual and/or electronic data review will be performed to identify possible data discrepancies. Manual and/or automatic queries will be created in the EDC system and will be issued to the site for appropriate response. Site staff will be responsible for resolving all queries in the database.

13.1.1. Paper Questionnaires

Data from paper forms may be collected from the site using fax and automatic character recognition. Paper forms and questionnaires are completed by the subject or a clinician and then sent via facsimile from the site. When the forms are received, each page is automatically scanned and indexed by intelligent character recognition (ICR) software, and uploaded into the EDC system.

13.1.2. Electronic Questionnaires

Questionnaires in electronic form may be collected directly using an electronic data collection platform at the clinical site (e.g. iPad). After completion by the subject or a clinician, data from the electronic questionnaires are transmitted directly into the EDC system.

13.1.3. Direct Data Upload

For quality assurance purposes and validation, technical data on the Boston Scientific Vercise DBS device, stored in the Boston Scientific Clinician Programmer, may be collected using direct data upload to a secure BSC server.

13.2. *Data Retention*

The Investigator or Investigational site will maintain, at the investigative site and in original format all essential study documents and source documentation that support the data collected on the study subjects in compliance with ICH/GCP guidelines.

Documents must be retained for at least 2 years after the last approval of a marketing application or until at least 2 years have elapsed since the formal discontinuation of the clinical investigation of the product or per the local regulatory requirements. These documents will be retained for a longer period of time by agreement with BSC or in compliance with other local regulations. It is BSC's responsibility to inform the Investigator when these documents no longer need to be maintained.

The Investigator will take measures to ensure that these essential documents are not accidentally damaged or destroyed. If for any reason the Investigator withdraws responsibility for maintaining these essential documents, custody must be transferred to an individual who will assume responsibility and BSC must receive written notification of this custodial change.

14. Study Assessments

14.1. *Adverse Events*

Adverse event evaluation will be conducted to identify adverse events occurring during the study and to classify them with regards to seriousness, severity, and relationship to either study procedure and/or study-device action. Additionally, an assessment of actions taken to address adverse events and their outcome will be conducted. Safety events will be reported as specified in Table 20-3. Only non-serious adverse events that are related to the device, stimulation, or procedure, and serious adverse events will be recorded. SAEs and USADEs will be collected from the time of informed consent through the end of the study.

14.2. *Burke-Fahn-Marsden Dystonia Rating Scale*

The Burke-Fahn-Marsden Dystonia Rating Scale (BFMDRS) (Burke et al., 1985) is composed of 2 clinician rated subscales: a movement subscale, based on patient examination, and a disability subscale, based on the patient's report of disability in activities of daily living. The movement subscale rates dystonia severity and provoking factors in 9 body areas, including eyes, mouth, speech and swallowing, neck, trunk, and both arms and legs. All items have a 5-point score. The provoking factor rates the relation of dystonia to action, from 0 (no dystonia at rest or with action) to 4 (dystonia at rest). The score obtained for eyes, mouth, and neck are each multiplied by 0.5, before being entered into the calculation of the total score, in order to down-weight them. The total movement BFMDRS sub-score is provided by the sum of the products of the provoking, severity, and weighting factors. The maximal total BFMDRS score is 120. The disability subscale is composed of 7 items for activities of daily living, such as speech, writing, feeding, eating, hygiene, dressing, and walking. These are rated on a 5-point score (with the exception of walking, which is rated on a 7-point score), providing a maximum disability sub-score of 30.

14.3. *Concomitant Medications*

All prescribed dystonia-related medications (including botox) will be collected throughout the study in order to obtain a full record of medication-related resource utilization. Information will include medication name, dates of prescription, dose, frequency, and route of administration.

14.4. *Clinical Global Impression of Change (CGI-C)*

Clinical Global Impression of Change (CGI-C) (Guy) is a 7-point scale that asks the clinician to assess how much the subject's condition has improved or worsened relative to the start of the study, prior to intervention. Clinicians rate the subject as: (1) very much improved; (2) much improved; (3) minimally improved; (4) no change; (5) minimally worse; (6) much worse; or (7) very much worse.

The CGI-C is conducted in the *stim on/meds on* condition

14.5. *Clinical Global Impression of Change - Subject (CGI-C: Sub)*

Clinical Global Impression of Change - Subject (CGI-C: Sub) is a 7-point scale that requires the subject to assess how much their condition has improved or worsened relative to the start of the study, prior to intervention. Subjects will rate themselves as: (1) very much improved; (2) much improved; (3) minimally improved; (4) no change; (5) minimally worse; (6) much worse; or (7) very much worse.

CGI-C: Sub will be assessed in the *stim on/meds on* condition.

14.6. *Clinical Global Impression of Change - Caregiver (CGI-C: Crg)*

Clinical Global Impression of Change - Caregiver (CGI-C: Crg) is a 7-point scale that requires the subject's caregiver to assess how much the subject's condition has improved or worsened relative to the start of the study, prior to intervention. The caregiver will rate the subjects are as: (1) very much improved; (2) much improved; (3) minimally improved; (4) no change; (5) minimally worse; (6) much worse; or (7) very much worse.

CGI-C: Crg will be assessed in the *stim on/meds on* condition as applicable when the caregiver is available.

14.7. *Global Dystonia Rating Scale (GDS)*

The Global Dystonia Rating scale (GDS) is dystonia severity scale in the 14 body areas. The investigator assess' the subject to rate of the 14 body areas with ratings from 0-10 (0 is no dystonia, 1 minimal, 5 moderate and 10 severe dystonia). There are no modifying ratings or weighting factors in the GDS. The total score is the sum of the scores for all the body areas. The maximal total score of the GDS is 140.

14.8. *Impedance Recordings (Optional)*

Technical data on the device impedance will be collected.

14.9. *Medical History*

The medical history will include relevant surgeries and chronic conditions with specific attention to:

- Intracranial surgery, stroke, seizure
- History of prior neuromodulation implants
- Psychiatric history
- Presence of fixed joint contractures and cervical myelopathy
- Reaction to materials (such as latex, metals) and history of foreign body reactions

A detailed history to characterize the patient's dystonia will also be collected

14.10. *Montreal Cognitive Assessment (MoCA)*

The Montreal Cognitive Assessment (Nasreddine, et al. 2005) is a tool used to assess cognitive impairment and assesses several cognitive domains including memory recall, attention, concentration, working memory, etc. The form is a 30-point test and typically takes about 10 minutes to complete.

14.11. *Programming Parameters*

Standard information regarding the programming parameters used to program the subject's IPG, as well as measurements taken using the device (e.g. contact impedances) will be collected from the Clinician Programmer (CP).

14.12. *Resource Utilization Inventory (RUI)*

Health resource utilization data will be collected including:

- Hospital visits (Not associated with an Adverse Event. Data on post-implant hospitalizations will be collected separately);
- Emergency room visits;
- Outpatient visits;
- Home care;
- Physician visits by specialty;
- Physiotherapy;
- Occupational therapy;
- Speech therapy;
- Equipment Use;

Site staff will administer this assessment to subjects. Subjects will also be asked to provide information about time lost from work and leisure since the last study visit. In addition, the typical time spent per day since the last study visit by caregivers assisting the subject with various ADLs including washing, feeding, toileting, mobility and supervision. Any visits to the Emergency Room/hospital and therapy will also be documented. For the baseline visit, subjects will be asked to provide information for the previous 3 months.

14.13. *Satisfaction with Treatment (SWT)*

The Satisfaction with Treatment questionnaire asks subjects directly about their level of satisfaction with the treatment they have been receiving in the study along several dimensions. Dimensions include symptom relief, side effects, device ease of use, and global satisfaction. For children under the age of 18 years, this questionnaire should be completed by the parent or guardian.

14.14. *Short Form Health Survey-36 (SF-36v2), and Short Form Health Survey-10 (SF-10v2)*

The SF-36™ (Ware & Sherbourne, 1992) is s a patient-reported multi-purpose health survey with 36 questions. This scale is used in subjects 18 years of age or older. It yields an 8-scale profile of functional health and well-being scores as well as psychometrically-based physical and mental health summary measures and a preference-based health utility index. Four of the eight scales are related to physical functioning, and four are related to mental functioning. Scales related to physical functioning are Physical Functioning (PF), Role-Physical (RP), Bodily Pain (BP), and General Health (GH), and the scales associated with mental functioning are Vitality (VT), Social Functioning (SF), Role-Emotional (RE) and mental health (MH).

The SF-10™ Health Survey for Children is a parent-completed survey that contains 10 questions adapted from the Child Health Questionnaire (CHQ). This test is specifically designed and will be utilized for children under the age of 18. The SF-10 provides coverage across a wide range of domains, and is scored to produce physical and psychosocial health summary measures. The survey provides a quick and efficient means to measure health status in cases where CHQ domain scores are not necessary.

14.15. *Toronto Western Spasmodic Torticollis Rating Scale (TWSTRS)*

The Toronto Western Spasmodic Torticollis Rating Scale (TWSTRS) was developed for assessment of cervical dystonia, and is the most widely used scale for cervical dystonia. It is composed of 3 subscales that measure symptom severity, disability, and pain. The severity scale, clinician-rated, is composed of 11 items that assess head movements, duration of symptoms, effects of sensory tricks, shoulder elevation and anterior displacement, range of motion, and time in neutral position; the maximal score is 35. The disability scale, patient-rated, comprises 6 items, including daily activities, work, reading, and driving; the maximal score is 30. The pain scale, patient-rated, comprises 3 items including severity, duration, and disability due to pain; the maximal score is 20. Each subscale is scored independently and a total TWSTRS score (from 0 to 85) is calculated. A training tape for clinicians is available for the severity scale.

15. Amendments

If a protocol revision is necessary which affects the rights, safety or welfare of the subject or scientific integrity of the data, an amendment is required. Appropriate Ethics Committee approvals of the revised protocol must be obtained prior to implementation.

16. Deviations

An investigator shall notify the sponsor and the reviewing IRB/EC of any deviation from the investigational plan to protect the life or physical well-being of a subject in an emergency, and those deviations which affect the scientific integrity of the clinical investigation. Such notice shall be given as soon as possible, but no later than 5 working days after the emergency occurred, or per prevailing local requirements, if sooner than 5 working days.

All deviations from the investigational plan, with the reason for the deviation and the date of occurrence, must be documented and reported to the sponsor using the EDC (Electronic Data Capturing) system. Sites may also be required to report deviations to the IRB/EC, per local guidelines and government regulations.

Deviations will be reviewed and evaluated on an ongoing basis and, as necessary, appropriate corrective and preventive actions (including notification, center re-training, or discontinuation) will be put into place by the sponsor.

17. Compliance

17.1. *Statement of Compliance*

This study will be conducted in accordance with ISO 14155: Clinical Investigation of Medical Devices for Human Subjects – Good Clinical Practice, ethical principles that have their origins in the Declaration of Helsinki, and pertinent individual country laws and regulations. The study shall not begin until the required approval/favorable opinion from the Ethics Committee(s) has been obtained. Any additional requirements imposed by the EC or regulatory authority shall be followed, if appropriate.

17.2. *Investigator Responsibilities*

The Principal Investigator of an investigational center is responsible for ensuring that the study is conducted in accordance with the Clinical Study Agreement, the investigational plan/protocol, ISO 14155, ethical principles that have their origins in the Declaration of Helsinki, any conditions of approval imposed by the reviewing IRB/EC, and prevailing local and/or country laws and/or regulations, whichever affords the greater protection to the subject.

The Principal Investigator's responsibilities include, but are not limited to, the following.

- Prior to beginning the study, sign the Investigator Agreement and Protocol Signature page documenting his/her agreement to conduct the study in accordance with the protocol.

- Provide his/her qualifications and experience to assume responsibility for the proper conduct of the study and that of key members of the center team through up-to-date curriculum vitae or other relevant documentation and disclose potential conflicts of interest, including financial, that may interfere with the conduct of the clinical study or interpretation of results.
- Make no changes in or deviate from this protocol, except to protect the life and physical well-being of a subject in an emergency; document and explain any deviation from the approved protocol that occurred during the course of the clinical investigation.
- Create and maintain source documents throughout the clinical study and ensure their availability with direct access during monitoring visits or audits; ensure that all clinical-investigation-related records are retained per requirements.
- Ensure the accuracy, completeness, legibility, and timeliness of the data reported to the sponsor in the CRFs and in all required reports.
- Record, report, and assess (seriousness and relationship to the device/procedure) every serious adverse event and observed device deficiency.
- Report to BSC, per the protocol requirements, all SAEs and device deficiencies that could have led to a SADE.
- Report to the IRB/EC and regulatory authorities any SAEs and device deficiencies that could have led to a SADE, if required by the national regulations or this protocol or by the IRB/EC, and supply BSC with any additional requested information related to the safety reporting of a particular event.
- Allow the sponsor to perform monitoring and auditing activities, and be accessible to the monitor and respond to questions during monitoring visits.
- Allow and support regulatory authorities and the IRB/EC when performing auditing activities.
- Ensure that informed consent is obtained in accordance with this protocol and local IRB/EC requirements.
- Provide adequate medical care to a subject during and after a subject's participation in a clinical study in the case of adverse events, as described in the Informed Consent Form (ICF).
- Inform the subject of the nature and possible cause of any adverse events experienced.
- As applicable, provide the subject with necessary instructions on proper use, handling, storage, and return of the investigational device when it is used/operated by the subject.

- Inform the subject of any new significant findings occurring during the clinical investigation, including the need for additional medical care that may be required.
- Provide the subject with well-defined procedures for possible emergency situations related to the clinical study, and make the necessary arrangements for emergency treatment, including decoding procedures for blinded/masked clinical investigations, as needed.
- Ensure that clinical medical records are clearly marked to indicate that the subject is enrolled in this clinical study.
- Ensure that, if appropriate, subjects enrolled in the clinical investigation are provided with some means of showing their participation in the clinical investigation, together with identification and compliance information for concomitant treatment measures (contact address and telephone numbers shall be provided).
- Inform, with the subject's approval or when required by national regulations, the subject's personal physician about the subject's participation in the clinical investigation.
- Make all reasonable efforts to ascertain the reason(s) for a subject's premature withdrawal from clinical investigation while fully respecting the subject's rights.
- Ensure that an adequate investigation site team and facilities exist and are maintained and documented during the clinical investigation.
- Ensure that maintenance and calibration of the equipment relevant for the assessment of the clinical investigation is appropriately performed and documented, where applicable.

17.2.1. **Delegation of Responsibility**

When specific tasks are delegated by an investigator, including but not limited to conducting the informed consent process, the investigator is responsible for providing appropriate training and adequate supervision of those to whom tasks are delegated. The investigator is accountable for regulatory violations resulting from failure to adequately supervise the conduct of the clinical study.

17.3. *Ethics Committee*

Prior to gaining Approval-to-Enroll status, the investigational center will provide to the sponsor documentation verifying that their EC is registered or that registration has been submitted to the appropriate agency, as applicable according to national/regulatory requirements.

A copy of the written EC and/or competent authority approval of the protocol (or permission to conduct the study) and Informed Consent Form, must be received by the sponsor before recruitment of subjects into the study and shipment of investigational product/equipment. Prior

approval must also be obtained for other materials related to subject recruitment or which will be provided to the subject.

Annual EC approval and renewals will be obtained throughout the duration of the study as required by local/country or EC requirements. Copies of the Investigator's reports and the EC continuance of approval must be provided to the sponsor.

17.4. *Sponsor Responsibilities*

All information and data sent to BSC concerning subjects or their participation in this study will be considered confidential by BSC. Only authorized BSC personnel or a BSC representative including Contract Research Organization (CRO) will have access to these confidential records. Authorized regulatory personnel have the right to inspect and copy all records pertinent to this study. Study data collected during this study may be used by BSC for the purposes of this study, publication, and to support future research and/or other business purposes. All data used in the analysis and reporting of this study will be without identifiable reference to specific subject name.

Boston Scientific will keep subjects' health information confidential in accordance with all applicable laws and regulations. Boston Scientific may use subjects' health information to conduct this research, as well as for additional purposes, such as overseeing and improving the performance of its device, new medical research and proposals for developing new medical products or procedures, and other business purposes. Information received during the study will not be used to market to subjects; subject names will not be placed on any mailing lists or sold to anyone for marketing purposes.

17.4.1. **Role of Boston Scientific Representatives**

Boston Scientific personnel can provide technical support to the investigator and other health care personnel (collectively HCP) as needed during implant, testing required by the protocol, and follow-ups. Support may include HCP training, addressing HCP questions, or providing clarifications to HCPs concerning the operation of BSC devices.

At the request of the investigator and while under investigator supervision, BSC personnel may operate equipment during implant or follow-up, assist with the conduct of testing specified in the protocol, and interact with the subject to accomplish requested activities. Typical tasks may include the following.

- Interrogating the device or programming device parameters to protocol specified settings
- Performing device diagnostic testing using a programmer to obtain thresholds and impedance measurements

- Clarifying device behavior, operation or diagnostic output as requested by the investigator or other health care personnel
- Assisting with the collection of study data from programmers and other equipment
- Entering data on study worksheets as long as the responsible HCP verifies and signs the completed worksheet.

In addition, BSC personnel may perform certain activities to ensure study quality. These activities may include the following.

- Observing testing or medical procedures to provide information relevant to protocol compliance
- Reviewing collected data and study documentation for completeness and accuracy

Boston Scientific personnel will not do the following.

- Practice medicine
- Provide medical diagnosis or treatment to subjects
- Discuss a subject's condition or treatment with a subject without the approval and presence of the HCP
- Independently collect critical study data (defined as primary or secondary endpoint data)
- Enter data in electronic data capture systems or on paper case report forms

17.5. *Insurance*

Where required by local/country regulation, proof and type of insurance coverage, by BSC for subjects in the study will be obtained.

18. Monitoring

Monitoring will be performed during the study to assess continued compliance with the protocol and applicable regulations. In addition, the monitor verifies that study records are adequately maintained, that data are reported in a satisfactory manner with respect to timeliness, adequacy, and accuracy, and that the Investigator continues to have sufficient staff and facilities to conduct the study safely and effectively. The Investigator/institution guarantees direct access to original source documents by Boston Scientific personnel, their designees, and appropriate regulatory authorities.

The study may also be subject to a quality assurance audit by Boston Scientific or its designees, as well as inspection by appropriate regulatory authorities. It is important that the Investigator

and relevant study personnel are available during on-site monitoring visits or audits and that sufficient time is devoted to the process.

18.1. *Monitoring Visits*

Details of the monitoring visits will be laid out in a Monitoring Plan.

A trained Boston Scientific Clinical Research Associate (CRA) or an appropriate delegated person shall initiate each investigation site to ensure that the Principal Investigator and, as applicable, his investigation site team:

- understand and accept the obligation to conduct the study according to the CIP and applicable regulations;
- have received and understood the requirements and contents of Manual of Instruction (DFU), Informed Consent Form and Case report Forms;
- have disclosed potential conflicts of interest, including financial, that interfere with the conduct of the clinical investigation or interpretation of results;
- have sufficient time and facilities and have access to an adequate number of appropriate subjects to conduct the study;
- have signed the Clinical Study Agreement.

In certain circumstances, an investigator meeting can be conducted instead of, or in addition to, the on-site initiation visit.

Monitoring visits will be conducted at regular intervals during the clinical investigation to assess the continued acceptability of the facilities, the continued compliance with the CIP and applicable regulations and the maintenance of adequate study records. During monitoring visits, the monitor may assess the case report forms submitted by the investigator with respect to timeliness, adequacy, and accuracy by reviewing source documents for a representative number of case reports when necessary. The Investigator Site File will also be reviewed for completeness, as specified in the Monitoring Plan.

A final monitoring visit will be conducted before study closure at each site in order to:

- reach the minimum monitoring level requested by the Monitoring Plan;
- ensure that all Adverse Events have been reported in the study database;
- solve any pending issues including missing data, unresolved queries in the study database and eventual corrections or incomplete Investigator Site File documentation.

For effective monitoring to take place, the investigator will allow the monitor designated by the sponsor direct access to review completed case report forms and clinical records. The investigator will agree to dedicate an adequate amount of time to monitoring.

18.2. *Securing Compliance*

In the event of repeated non-compliance, as determined by Boston Scientific clinical management, a Boston Scientific CRA or delegated representative will attempt to secure compliance by one or more of the following:

- Visiting the investigator;
- Calling the investigator;
- Corresponding with the investigator;
- Requesting corrective actions through a Corrective Action Plan.

If an investigator is found to be repeatedly non-compliant with the signed agreement, the CIP or any other conditions of the study, Boston Scientific will either secure compliance or, at its sole discretion, suspend enrollment or terminate the investigator’s participation in the study. In the event of termination of investigator participation, study devices will be returned to Boston Scientific unless this action would jeopardize the rights, safety or welfare of the patient (s).

19. Potential Risks and Benefits

Based on clinical experience with the commercially available DBS therapy for dystonia, there are anticipated risks that may be associated with the use of the Vercise™ System. These include risks related to the surgical implant procedure, device hardware, stimulation (neuropsychiatric, neurocognitive, motor, other), and/or side effects of medications.

19.1. *Anticipated Adverse Device Effects*

The following anticipated adverse device effects (ADE) have been identified for use of Deep Brain Stimulation for treatment of dystonia. These include potential effects due to presence of the device, whether it is “on” or “off,” effects due to use of stimulation, and effects related to the device during a study surgical procedure. Note that some of these stimulation-related symptoms may be resolved or reduced by current steering, changing stimulation parameters, or by surgical repositioning of the lead:

- Allergic, immune, or inflammatory response;
- CSF leak;
- Death, including suicide;
- Electric shock due to misuse of charger base station;
- Failure or malfunction of any of the device components or the battery, including but not limited to lead or extension breakage, hardware malfunctions, loose connections, electrical shorts or open circuits and lead insulation breaches, whether or not this requires explant and/or re-implantation;
- Hemorrhagic or ischemic stroke, immediate or delayed, which could result in temporary or permanent neurologic deficits such as muscle weakness, paralysis or aphasia;
- Implant site complications such as pain, poor healing, wound reopening;
- Infection;
- Injury to tissues adjacent to implant or within surgical field, such as blood vessels, peripheral nerves, brain (including pneumocephalus), or pleura (including pneumothorax);
- Interference from external electromagnetic sources;
- Lead, extension (including extension header) and IPG erosion or migration;
- Loss of adequate stimulation;
- Cognitive impairment such as attention or executive deficits, memory disturbances, confusion, or dysphasia;
- Psychiatric disturbances such as anxiety, depression, apathy, mania, insomnia, suicide, or suicidal ideation or attempts;
- Motor problems such as paresis, weakness, incoordination, restlessness, muscle spasms, postural and gait disorders, tremor, worsened dystonia, or dyskinesias, and falls or injuries resulting from these problems;
- Musculoskeletal stiffness;
- Overstimulation or undesirable sensations, such as paresthesia, transient or persistent;
- Neurosurgery risks, including unsuccessful implant, exposure to blood borne pathogens;
- Pain, headache or discomfort, transient or persistent, including symptoms due to neurostimulation;

- Poor initial lead location, which may lead to ineffective therapy, side effects, or a surgical revision;
- Radiation exposure due to imaging (CT, fluoroscopy x-ray);
- Seizures;
- Sensory changes (e.g. changes in hearing, taste, or other senses);
- Seroma, edema or hematoma;
- Skin irritation or burns at IPG site;
- Speech or swallowing problems such as dysarthria or dysphagia, as well as complications of dysphagia such as aspiration pneumonia;
- Symptoms in other systems, such as tachycardia, sweating, fever, dizziness, changes in renal function, urinary retention, sexual effects, nausea, bowel retention, bloating;
- Thrombosis;
- Visual disturbances or periorbital symptoms, such as diplopia, eyelid movement difficulty, oculomotor difficulties or other visual field effects;
- Weight changes.

19.2. *Contraindications*

Strong electromagnetic fields can potentially turn the device off, cause temporary unpredictable changes in stimulation, or interfere with the remote control communication.

Subjects should avoid or exercise care around:

- Theft detectors, such as those used at entrances/exits of department stores, libraries, and other public establishments: These can cause the device to turn off or cause an uncomfortable or jolting sensation. If the subject must proceed through the detector, he/she should proceed with caution, ensuring to move through the center of the detector as quickly as possible;
- Power lines, power generators, electric steel furnaces, arc welders, and large magnetized stereos: These may turn the device off and/or cause uncomfortable or jolting sensation;
- Security screeners, such as those used in Airport Security or at entrances to government buildings, including hand-held scanners: These can cause the device to turn off or cause an uncomfortable or jolting sensation. It is recommended that subjects request assistance to bypass the screener. If they must proceed, then they should be advised to turn off the

device, proceed with caution, ensuring to move quickly through the security screener and staying as far from the screener as allowable.

19.3. *Anticipated Adverse Events*

The following anticipated adverse events (AE) have been identified for this study. These include risks that could occur due to the implant procedure, revision of system, explant, or replacement of device:

- Allergic, immune system response or reaction;
- Anesthesia/neurosurgery risks, including unsuccessful implant, exposure to blood borne pathogens;
- CSF leak;
- Death, including suicide;
- Embolism, including air embolism and pulmonary embolism;
- Hemorrhagic or ischemic stroke, immediate or delayed, which could result in temporary or permanent neurologic deficits such as muscle weakness, paralysis or aphasia;
- Implant site complications such as pain, poor healing, wound reopening;
- Infection;
- Injury to tissues adjacent to implant or within surgical field, such as blood vessels, peripheral nerves, brain (including pneumocephalus), or pleura (including pneumothorax);
- Cognitive impairment such as attention or executive deficits, memory disturbances, confusion, or dysphasia;
- Psychiatric disturbances such as anxiety, depression, apathy, mania, insomnia, suicide, or suicidal ideation or attempts;
- Motor problems such as paresis, weakness, incoordination, restlessness, muscle spasms, postural and gait disorders, tremor, worsened dystonia, or dyskinesias, and falls or injuries resulting from these problems;
- Musculoskeletal stiffness;
- Pain, headache or discomfort, transient or persistent;
- Poor initial lead location, which may lead to ineffective therapy, side effects, or a surgical revision;

- Radiation exposure due to imaging (CT, fluoroscopy x-ray);
- Seizures;
- Sensory changes (e.g. changes in hearing, taste, or other senses);
- Seroma, edema or hematoma;
- Speech or swallowing problems such as dysarthria or dysphagia, as well as complications of dysphagia such as aspiration pneumonia;
- Symptoms in other systems, such as tachycardia, sweating, fever, dizziness, changes in renal function, urinary retention, sexual effects, nausea, bowel retention, bloating;
- Thrombosis;
- Visual disturbances or periorbital symptoms, such as diplopia, eyelid movement difficulty, oculomotor difficulties or other visual field effects.

19.4. *Risks Associated with the Study Device(s)*

The study device has a rechargeable battery and certain risks are associated with this functionality. These are specific to the study device since most of the devices available in the market have non-rechargeable (primary cell) batteries. The risks specific to rechargeable devices include:

- Additional subject burden: If the IPG battery depletes too frequently, it may require frequent charging or additional intervention including explant;
- Burn: Due to charger misuse;
- Inadequate stimulation: Failure to recharge the device correctly or adequately can result in inadequate stimulation. One way this could happen is if the subject incorrectly interprets the battery status icon on the remote control;
- Pain: If the subject uses the adhesive patch to hold the charger in place during charging, it is possible he/she may have difficulty removing the patch and experience pain;
- Allergic reaction: Due to incompatibility of the adhesive (on the patch used for charging) with the subject's skin;
- Surgical repositioning of IPG: The IPG may need to be repositioned if it is located too deep, resulting in the subject being unable to charge or hear the charger beep which indicates end of charging.

These additional risks are mitigated by the fact that the rechargeable study device is expected to avoid the risk of additional surgery for replacement of a depleted primary cell battery.

19.5. *Risks associated with Participation in the Clinical Study*

The following risks maybe associated with subjects' participation in the clinical study:

- Subjects may find it difficult, uncomfortable, or tiresome to complete study visits and questionnaires;
- Subjects with postural instability or gait disturbances either due to dystonia or as a side effect of DBS may be at a risk of falling while standing or walking for certain study assessments.

19.6. *Possible Interactions with Concomitant Medical Treatments*

Medications used to treat dystonia (anticholinergics, GABA agonists or modulators, anticonvulsants, dopaminergics, etc.) may be adjusted in response to effects of DBS. Medication changes may produce unwanted symptoms during the adjustment period.

19.7. *Risk Minimization Actions*

Additional risks may exist for subjects participating in the clinical study (as described above). However, these risks can be minimized through compliance with this protocol, performing procedures in the appropriate hospital environment, adherence to subject selection criteria, close monitoring of the subject's physiological status during research procedures and/or follow-ups and by promptly supplying BSC with all pertinent information required by this protocol.

The Vercise™ device used in this study is similar to Medtronic's Activa Therapy device that has an extensive history of use in the treatment of dystonia. Based on clinical experience with the Medtronic therapy, the anticipated risks associated with the use of the Vercise™ device are predicted to be acceptable compared to the expected benefits in the reduction of dystonia symptoms. The treatment is reversible in that the device may be turned off or explanted at any time for any reason.

All efforts will be made to minimize the aforementioned potential risks using the following approaches:

- Selection of Investigators (neurologists and neurosurgeons) who are experienced and skilled in the treatment of patients with dystonia as per BSC's site selection and qualification procedures;
- Clearly defined inclusion and exclusion criteria that ensure only appropriate patients are enrolled;
- Ensuring that treatment and follow-up of patientts is consistent with current medical practice;

- Safety review processes by Boston Scientific and Independent Data Reviewer;
- Monitoring visits to investigational sites.

19.8. *Anticipated Benefits*

High frequency stimulation of deep brain structures has been used since the 1980s for the treatment of motor symptoms associated with Parkinson's disease. DBS was approved for Medtronic as a Humanitarian Exemption for use in dystonia in 2003. Many studies have measured the risks and benefits associated with this type of treatment, and considerable reduction in symptoms has been observed. It is possible that some individual patients may experience no direct benefit from participation in this study. However, based on the success of the Medtronic's Activa Therapy, it is anticipated that most patients will experience improvement in dystonia symptoms. The small size, , wide parameter ranges, and precise control of stimulation of the Vercise™ system are anticipated to be perceived as benefits.

19.9. *Risk to Benefit Rationale*

The purpose of this study is to compile characteristics of real-world outcomes for Boston Scientific Corporation's commercially approved Vercise System for deep brain stimulation (DBS), when used according to the applicable Directions for Use. The mechanism of action of the Vercise System is the same as other commercially available DBS systems. Based on the clinical data for the approved Medtronic's Activa DBS System, the risk-to-benefit ratio for BSC Vercise™ DBS System is within reason for foreseeable risks. However, studies do not always predict all side effects that may be experienced. Observation and follow-up of all patients is required as outlined in the protocol.

20. Safety Reporting

20.1. *Reportable Events by investigational site to Boston Scientific*

It is the responsibility of the investigator to assess and report to BSC any event which occurs in any of following categories:

- All Serious Adverse Events
- All Device Deficiencies
- Unanticipated Adverse Device Effects/Unanticipated Serious Adverse Device Effects
- New findings/updates in relation to already reported events

- All Device Related Adverse Events
- All Study Procedure Related Adverse Events

When possible, the medical diagnosis should be reported as the Event Term instead of individual symptoms.

If it is unclear whether or not an event fits one of the above categories, or if the event cannot be isolated from the device or procedure, it should be submitted as an adverse event and/or device deficiency.

Any AE event required by the protocol, experienced by the study subject after informed consent and once considered enrolled in the study (as defined in study subject classification section), whether during or subsequent to the procedure, must be recorded in the eCRF.

Underlying diseases are not reported as AEs unless there is an increase in severity of frequency during the course of the investigation. For centers in Austria cancer must always be reported as a Serious Adverse Event. Death should not be recorded as an AE, but should only be reflected as an outcome of ONE (1) specific SAE (see Table 20.2-1 for AE definitions).

Refer to Section 19 for the known risks associated with the study device(s).

20.2. *Definitions and Classifications*

Adverse event definitions are provided in Table 20.2-1.

Table 20.2-1: Adverse Event Definitions

Term	Definition
Adverse Event (AE)<br><br>*Ref: ISO 14155*<br><br>*Ref: MEDDEV 2.7/3*	Any untoward medical occurrence, unintended disease or injury, or any untoward clinical signs (including an abnormal laboratory finding) in subjects, users or other persons, whether or not related to the investigational medical device.<br><br>NOTE 1: This includes events related to the investigational medical device or comparator.<br><br>NOTE 2: This definition includes events related to the procedures involved (any procedure in the clinical investigation plan).<br><br>NOTE 3: For users or other persons, this definition is restricted to events related to the investigational medical device.
Adverse Device Effect (ADE)<br><br>*Ref: ISO 14155*<br><br>*Ref: MEDDEV 2.7/3*	Adverse event related to the use of an investigational medical device<br><br>NOTE 1: This definition includes any adverse event resulting from insufficient or inadequate instructions for use, the deployment, the implantation, the installation, the operation, or any malfunction of the investigational medical device.<br><br>NOTE 2: This definition includes any event resulting from use error or from

Table 20.2-1: Adverse Event Definitions

Term	Definition
	intentional misuse of the investigational medical device.
Serious Adverse Event (SAE)<br><br>*Ref: ISO 14155*<br><br>*Ref: MEDDEV 2.7/3*	Note: This definition meets the reporting objectives and requirements of ISO 14155 and MEDDEV 2.7/3.<br>Adverse event that:<br>• Led to death,<br>• Led to serious deterioration in the health of the subject, as defined by either:<br>o a life-threatening illness or injury, or<br>o a permanent impairment of a body structure or a body function, or<br>o in-patient or prolonged hospitalization of existing hospitalization, or<br>o medical or surgical intervention to prevent life-threatening illness or injury or permanent impairment to a body structure or a body function<br>• Led to fetal distress, fetal death, or a congenital abnormality or birth defect.<br>**NOTE 1**: Planned hospitalization for a pre-existing condition, or a procedure required by the clinical investigational plan, without serious deterioration in health, is not considered a serious adverse event.
Serious Adverse Device Effect (SADE)<br><br>*Ref: ISO 14155*<br><br>*Ref: MEDDEV 2.7/3*	Adverse device effect that has resulted in any of the consequences characteristic of a serious adverse event.
Unanticipated Serious Adverse Device Effect (USADE)<br><br>*Ref: ISO 14155*<br><br>*Ref: MEDDEV 2.7/3*	Serious adverse device effect which by its nature, incidence, severity, or outcome has not been identified in the current version of the risk analysis report.<br>**NOTE 1**: Anticipated serious adverse device effect (ASADE) is an effect which by its nature, incidence, severity or outcome has been identified in the risk analysis report.
Device Deficiency	A device deficiency is any inadequacy of a medical device with respect to its identity, quality, durability, reliability, safety or performance.<br>**NOTE 1**: Device deficiencies include malfunctions, misuse or use errors, and

Table 20.2-1: Adverse Event Definitions

Term	Definition
Ref: ISO 14155<br><br>*Ref: MEDDEV 2.7/3*	inadequate labeling.

Abbreviations: EC=Ethics Committee; IRB=Institutional Review Board

Underlying diseases are not reported as AEs unless there is an increase in severity or frequency during the course of the investigation. Death should not be recorded as an AE, but should only be reflected as an outcome of a specific SAE (see Table 20-1 for AE definitions).

Refer to Section 19 for the known risks associated with the study device(s).

NOTES:

- For the purposes of this study, hospitalization is defined as any in-patient admission.
- Hospitalizations occurring for the purpose of performing a planned procedure as per routine/standard of care such as programming sessions or standard evaluation, staged implant procedures or rehabilitation are NOT to be reported as a SAE.
- Any complications or adverse events that occur during an elective/planned hospitalization, should be reported if they meet the protocol specified definitions. However, the original elective/planned hospitalization(s) should not be reported as an SAE.
- Sensations or side effects that occur during programming should not be reported as AEs. However, persistent unpleasant sensations or side effects that occur after the completion of programming will be reported.
- Ineffective therapy (e.g. lack of efficacy, lack or decrease of therapeutic response) will not be collected as an adverse event, since failure to achieve therapeutic response is an issue of efficacy, not safety. No AE of “Ineffective Therapy” should be reported when using the expanded parameter range for purposes of improving ineffective therapy. However, AEs due to sudden loss of efficacy (e.g. loss of stimulation leading to a fall or injury) will be collected.
- The subject’s dystonia symptoms will not be collected as AEs, unless they have worsened beyond baseline and beyond the expected disease progression over time. This worsening event could occur in the context of a device malfunction or with a properly-functioning device as determined by investigator).

- Device migration will not be collected as an adverse event. However, an AE that results from the device/lead migration should be reported as an AE. Device migration should be reported as a device deficiency in the electronic database.
- In the case of death, efforts should be made to perform an autopsy. If an autopsy is performed the study device should be explanted and returned to BSC. If no autopsy is performed, attempt should be made to explant the device, if feasible, and the device returned to BSC. As well, the device should be explanted before cremation due to risk of battery explosion when exposed to extreme heat. A copy of the death records and an autopsy report (if available) should also be sent to Boston Scientific as soon as possible, provided that such activities do not violate patient consent and patient data confidentiality.

20.3. *Relationship to Study Device(s)*

The Investigator must assess the relationship of the AE to the study device and/or study procedure as described in Table 20.3-1

.

Table 20.3-1: Criteria for Assessing Relationship of Study Device or Procedure to Adverse Event

Classification	Description
**Not Related**	Relationship to the device or procedures can be excluded when:<br>- the event is not a known side effect of the product category the device belongs to or of similar devices and procedures;<br>- the event has no temporal relationship with the use of the investigational device or the procedures;<br>- the serious event does not follow a known response pattern to the medical device (if the response pattern is previously known) and is biologically implausible;<br>- the discontinuation of medical device application or the reduction of the level of activation/exposure - when clinically feasible – and reintroduction of its use (or increase of the level of activation/exposure), do not impact on the serious event;<br>- the event involves a body-site or an organ not expected to be affected by the device or procedure; the serious event can be attributed to another cause (e.g. an underlying or concurrent illness/ clinical condition, an effect of another device, drug, treatment or other risk factors);<br>- the event does not depend on a false result given by the investigational device used for diagnosis, when applicable; harms to the subject are not clearly due to use error;<br>- In order to establish the non-relatedness, not all the criteria listed above might be met at the same time, depending on the type of device/procedures and the serious event.
**Unlikely Related**	The relationship with the use of the device seems not relevant and/or the event can be reasonably explained by another cause, but additional information may be obtained.
**Possibly Related**	The relationship with the use of the investigational device is weak but cannot be ruled out completely. Alternative causes are also possible (e.g. an underlying or concurrent illness/ clinical condition or/and an effect of another device, drug or treatment). Cases were relatedness cannot be assessed or no information has been obtained should also be classified as possible.
**Probably Related**	The relationship with the use of the investigational device seems relevant and/or the event cannot reasonably explained by another cause, but additional information may be obtained.
**Causal Relationship**	The serious event is associated with the investigational device or with procedures beyond reasonable doubt when:<br>- the event is a known side effect of the product category the device belongs to or of similar devices and procedures;<br>- the event has a temporal relationship with investigational device use/application or procedures;<br>- the event involves a body-site or organ that<br>o the investigational device or procedures are applied to;<br>o the investigational device or procedures have an effect on;<br>- the serious event follows a known response pattern to the medical device (if the response pattern is previously known);<br>- the discontinuation of medical device application (or reduction of the level of

Table 20.3-1: Criteria for Assessing Relationship of Study Device or Procedure to Adverse Event

Classification	Description
	activation/exposure) and reintroduction of its use (or increase of the level of activation/exposure), impact on the serious event (when clinically feasible);<br>- other possible causes (e.g. an underlying or concurrent illness/ clinical condition or/and an effect of another device, drug or treatment) have been adequately ruled out;<br>- harm to the subject is due to error in use;<br>- the event depends on a false result given by the investigational device used for diagnosis, when applicable;<br>- In order to establish the relatedness, not all the criteria listed above might be met at the same time, depending on the type of device/procedures and the serious event.

20.4. *Investigator Reporting Requirements*

The communication requirements for reporting to BSC are described in Table 20.4-1

Table 20.4-1: Investigator Reporting Requirements

Event Classification	Communication Method	Communication Timeline post market studies*<br>(MEDDEV 2.12/2 : GUIDELINES ON A MEDICAL DEVICE VIGILANCE SYSTEM)
Unanticipated Serious Adverse Device Effect (USADE)**	Complete AE eCRF page with all available new and updated information	• Within 1 business day of first becoming aware of the event.<br>• Terminating at the end of the study<br>• Includes all events related to hardware, stimulation and procedure
Serious Adverse Event	Complete AE eCRF page with all available new and updated information	• Within 10 business days of first becoming aware of the event or as per local/regional regulations.<br>• For Austria: within 2 business days of becoming aware of the event<br>• Reporting required through the end of the study
	Provide all relevant source documentation (unidentified) for reported event upon request of the	• When documentation is available

Table 20.4-1: Investigator Reporting Requirements

**Event Classification**	**Communication Method**	**Communication Timeline post market studies***<br><br>(MEDDEV 2.12/2 : GUIDELINES ON A MEDICAL DEVICE VIGILANCE SYSTEM)
	sponsor	
Serious Adverse Device Effects**	Complete AE eCRF page with all available new and updated information<br><br>Provide all relevant source documentation (unidentified) for reported event	• Within 2 business days of first becoming aware of the event or as per local/regional regulations.<br>• Reporting required through the end of the study for all events related to hardware, stimulation and procedure<br>• When documentation is available
Adverse Device Effects**	Complete AE eCRF page, which contains such information as date of AE, treatment of AE resolution, assessment of seriousness and relationship to the device	• In a timely manner (e.g. recommended within 30 business days) after becoming aware of the information<br>• Reporting required through end of the study for all events related to hardware, stimulation and procedure
Device Deficiencies (including but not limited to failures, malfunctions, and product nonconformities)<br><br>Note: Any Investigational Device Deficiency that might have led to a serious adverse event if a) suitable action had not been taken or b) intervention had not been made or c) if circumstances had been less fortunate is considered a reportable event.	Complete Device Deficiency CRF with all available new and updated information	• Within 2 business day of first becoming aware of the event.<br>• Reporting required through the end of the study

Abbreviations: AE=adverse event; CRF=case report form; USADE=unanticipated serious adverse device effect

*Please note that post-market studies are clinical studies where the medical devices used in the study bear the

Table 20.4-1: Investigator Reporting Requirements

Event Classification	Communication Method	Communication Timeline post market studies* (MEDDEV 2.12/2 : GUIDELINES ON A MEDICAL DEVICE VIGILANCE SYSTEM)

regulatory approval and are used for the same approved indications.
**Includes events that are related to hardware, stimulation and procedure

The investigator must report Adverse Device Effects, Serious Adverse Events (regardless of relationship to device hardware, stimulation and/or procedure), Unanticipated Serious Adverse Device Effects, and Device Deficiencies for each subject from the time of Information Consent through the end of study participation. AEs and Device Deficiencies may be reported via phone, fax or email if the electronic data capture (EDC) system is unavailable. The paper AE Notification Form or Device Deficiency Notification Form should be used to report AEs and/or device deficiencies during this time.

The Investigator must assess the potential relationship of all adverse events to the study device and/or to study procedures.

- Adverse events must be assessed according to their relationship to one of the following categories:
  - **Device Hardware-Related AEs:** AEs that can reasonably (i.e. Unlikely, Possibly, Probably and Causally Related) be attributed to the mere physical presence of the device or to deficiency of the device (i.e., an allergic response to device materials).
  - **Stimulation-Related AEs:** AEs that can reasonably (i.e. Unlikely, Possibly, Probably and Causally Related) be attributed to the effects of stimulation. A relationship to stimulation may be determined by demonstrating a predictable response to the alternating between stimulation-on and stimulation-off settings. However, a relationship to stimulation may also be reported without demonstrating a predictable response to the alternating between the stimulation-on and stimulation-off settings if in the opinion of the Investigator the AE is potentially related to stimulation.
  - **Procedure Related AEs:** AEs that can reasonably (i.e. Unlikely, Possibly, Probably and Causally Related) be attributed to a study protocol required procedure.

20.5. *Boston Scientific Device Deficiencies*

All device deficiencies (including but not limited to failures, malfunctions, use errors, product nonconformities, and labeling errors) will be documented and reported to BSC. If possible, the device(s) should be returned to BSC for analysis. Instructions for returning the investigational device(s) will be provided. If it is not possible to return the device, the investigator should document why the device was not returned and the final disposition of the device. Device failures and malfunctions should also be documented in the subject's medical record.

All device deficiencies whether or not associated with an adverse event should be reported on the device deficiency eCRF.

Device deficiencies (including but not limited to failures, malfunctions, and product nonconformities) are not to be reported as adverse events. However, if an adverse event is associated with a device deficiency, that specific event should be recorded in the AE eCRF.

If a device deficiency could have led to a serious adverse event if A) suitable action had not been taken or B) intervention had not been made or C) if circumstances had been less fortunate is considered a reportable event.

20.6. *Reporting to Regulatory Authorities / ECs / Investigators*

BSC is responsible for reporting adverse event information to all participating investigators and regulatory authorities, as applicable.

The Sponsor, Investigator, or Site must notify the IEC of any USADEs, SADEs, SAEs, and other events as applicable according to local reporting requirements. A copy of the Investigator's reports and other relevant reports (if applicable) to the IEC must be provided to BSC in accordance with the local requirements.

Boston Scientific Corporation will notify all participating study centers if SAEs/SADEs occur which imply a possible increase in the anticipated risk of the procedure or use of the device or if the occurrence of certain SAEs/SADEs demands changes to the protocol or the conduct of the study in order to further minimize the unanticipated risks.

21. Informed Consent

Subject participation in this clinical study is voluntary. Informed Consent is required from all subjects or their legally authorized representative. The Investigator is responsible for ensuring that Informed Consent is obtained prior to the use of any investigational devices, study-required procedures and/or testing, or data collection.

The obtaining and documentation of Informed Consent must be in accordance with the principles of the Declaration of Helsinki, ISO 14155, any applicable national regulations, and local Ethics Committee and/or Regulatory authority body, as applicable. The ICF must be approved by the center's IRB/EC, or central IRB, if applicable.

Boston Scientific will provide a study-specific template of the ICF to investigators participating in this study. The ICF template may be modified to meet the requirements of the investigative center's IRB/EC. Any modification requires approval from BSC prior to use of the form. The ICF must be in a language understandable to the subject and if needed, BSC will assist the center in obtaining a written consent translation. Translated consent forms must also have IRB/EC approval prior to their use. Privacy language shall be included in the body of the form or as a separate form as applicable.

The process of obtaining Informed Consent shall:

- be conducted by the Principal Investigator or designee authorized to conduct the process,
- include a description of all aspects of the clinical study that are relevant to the subject's decision to participate throughout the clinical study,
- avoid any coercion of or undue influence of subjects to participate,
- not waive or appear to waive subject's legal rights,
- use native language that is non-technical and understandable to the subject or his/her legal representative,
- provide ample time for the subject to consider participation and ask questions if necessary,
- ensure important new information is provided to new and existing subjects throughout the clinical study.

The ICF shall always be signed and personally dated by the subject or legal representative and by the investigator or an authorized designee responsible for conducting the informed consent process. If a legal representative signs, the subject shall be asked to provide informed consent for continued participation as soon as his/her medical condition allows. The original signed ICF will be retained by the center and a copy of the signed and dated document and any other written information must be given to the person signing the form.

Failure to obtain subject consent will be reported by BSC to the applicable regulatory body according to their requirements (e.g., FDA requirement is within 5 working days of learning of such an event). Any violations of the informed consent process must be reported as deviations to the sponsor and local regulatory authorities (e.g. IRB/EC), as appropriate.

If new information becomes available that can significantly affect a subject's future health and medical care, that information shall be provided to the affected subject(s) in written form via a revised ICF or, in some situations, enrolled subjects may be requested to sign and date an addendum to the ICF. In addition to new significant information during the course of a study, other situations may necessitate revision of the ICF, such as if there are amendments to the protocol, a change in Principal Investigator, administrative changes, or following annual review by the IRB/EC. The new version of the ICF must be approved by the IRB/EC. Boston Scientific approval is required if changes to the revised ICF are requested by the center's IRB/EC. The IRB/EC will determine the subject population to be re-consented.

22. Committees

22.1. *Safety Monitoring Process*

To promote early detection of safety issues, the Medical Director will provide evaluations of safety events. Success of this program requires dynamic collection of unmonitored data as soon as the event is reported. This is expedited through BSC Safety Office, which is responsible for coordinating the collection of information for the subject dossier from the centers and core laboratories. During regularly scheduled monitoring visits, clinical research monitors will support the dynamic reporting process through their review of source document information.

23. Suspension or Termination

23.1. *Premature Termination of the Study*

Boston Scientific Corporation reserves the right to terminate the study at any stage but intends to exercise this right only for valid scientific or administrative reasons and reasons related to protection of subjects. Investigators, associated IRBs/ECs, and regulatory authorities, as applicable, will be notified in writing in the event of study termination.

23.2. *Criteria for Premature Termination of the Study*

Possible reasons for premature study termination include, but are not limited to, the following.

- The occurrence of unanticipated serious adverse device effects that present a significant or unreasonable risk to subjects enrolled in the study.
- An enrollment rate far below expectation that prejudices the conclusion of the study.

- A decision on the part of Boston Scientific to suspend or discontinue development of the device.

23.3. *Termination of Study Participation by the Investigator or Withdrawal of IRB/ EC Approval*

Any investigator, or IRB/ EC may discontinue participation in the study or withdrawal approval of the study, respectively, with suitable written notice to Boston Scientific. Investigators, associated IRBs/ECs, and regulatory authorities, as applicable, will be notified in writing in the event of these occurrences.

23.4. *Requirements for Documentation and Subject Follow-up*

In the event of premature study termination a written statement as to why the premature termination has occurred will be provided to all participating centers by Boston Scientific. The IRB/EC and regulatory authorities, as applicable, will be notified. Detailed information on how enrolled subjects will be managed thereafter will be provided.

In the event an IRB or EC terminates participation in the study, participating investigators, associated IRBs/ECs, and regulatory authorities, as applicable, will be notified in writing. Detailed information on how enrolled subjects will be managed thereafter will be provided by Boston Scientific.

In the event an investigator terminates participation in the study, study responsibility will be transferred to a co-investigator, if possible. In the event there are no opportunities to transfer investigator responsibility; detailed information on how enrolled subjects will be managed thereafter will be provided by Boston Scientific.

The investigator must return all documents and investigational product to Boston Scientific, unless this action would jeopardize the rights, safety, or welfare of the subjects.

23.5. *Criteria for Suspending/Terminating a Study Center*

Boston Scientific Corporation reserves the right to stop the inclusion of subjects at a study center at any time after the study initiation visit if no subjects have been enrolled for a period beyond 6 months after center initiation, or if the center has multiple or severe protocol violations/noncompliance without justification and/or fails to follow remedial actions.

In the event of termination of investigator participation, the EC and regulatory authorities, as applicable, should be notified. All subjects enrolled in the study at the center will continue to be followed per the study protocol. The Principal Investigator at the center must make provision for these follow-up visits unless BSC notifies the investigational center otherwise.

24. Publication Policy

In accordance with the Corporate Policy on the Conduct of Human Subject Research, BSC requires disclosure of its involvement as a sponsor or financial supporter in any publication or presentation relating to a BSC study or its results. In accordance with the Corporate Policy for the Conduct of Human Subject Research, BSC will submit study results for publication (regardless of study outcome) following the conclusion or termination of the study. Boston Scientific Corporation adheres to the Contributor ship Criteria set forth in the Uniform Requirements of the International Committee of Medical Journal Editors (ICMJE; http://www.icmje.org). In order to ensure the public disclosure of study results in a timely manner, while maintaining an unbiased presentation of study outcomes, BSC personnel may assist authors and investigators in publication preparation provided the following guidelines are followed.

- All authorship and contributor ship requirements as described above must be followed.
- BSC involvement in the publication preparation and the BSC Publication Policy should be discussed with the Coordinating Principal Investigator(s) and/or Executive/Steering Committee at the onset of the project.

The First and Senior authors are the primary drivers of decisions regarding publication content, review, approval, and submission.

25. Bibliography

1. Andrews, C., Aviles-Olmos, I., Hariz, M., & Foltynie, T. (2010). Which patients with dystonia benefit from deep brain stimulation? A metaregression of individual patient outcomes. *J Neurol Neurosurg Psychiatry, 81*(12), 1383.
2. Borggraefe, I., Mehrkens, J. H., Telegravciska, M., Berweck, S., Botzel, K., & Heinen, F. (2010). Bilateral pallidal stimulation in children and adolescents with primary generalized dystonia--report of six patients and literature-based analysis of predictive outcomes variables. *Brain Dev, 32*(3), 223.
3. Burke, R. E., Fahn, S., Marsden, C. D., Bressman, S. B., Moskowitz, C., & Friedman, J. (1985). Validity and reliability of a rating scale for the primary torsion dystonias. *Neurology, 35*(1), 73.
4. Defazio, G. (2010). The epidemiology of primary dystonia: current evidence and perspectives. *Eur J Neurol, 17 Suppl 1*, 9.

5. Deuschl, G., Schade-Brittinger, C., Krack, P., Volkmann, J., Schafer, H., Botzel, K., Voges, J. (2006). A randomized trial of deep-brain stimulation for Parkinson's disease. *N Engl J Med, 355*(9), 896.

6. Egidi, M., Franzini, A., Marras, C., Cavallo, M., Mondani, M., Lavano, A., Lanotte, M. (2007). A survey of Italian cases of dystonia treated by deep brain stimulation. *J Neurosurg Sci, 51*(4), 153.

7. Follett, K. A., Weaver, F. M., Stern, M., Hur, K., Harris, C. L., Luo, P., Reda, D. J. (2010). Pallidal versus subthalamic deep-brain stimulation for Parkinson's disease. *N Engl J Med, 362*(22), 2077.

8. Fraix, V., Houeto, J. L., Lagrange, C., Le Pen, C., Krystkowiak, P., Guehl, D., Pollak, P. (2006). Clinical and economic results of bilateral subthalamic nucleus stimulation in Parkinson's disease. *J Neurol Neurosurg Psychiatry, 77*(4), 443.

9. Gruber, D., Trottenberg, T., Kivi, A., Schoenecker, T., Kopp, U. A., Hoffmann, K. T., Kupsch, A. (2009). Long-term effects of pallidal deep brain stimulation in tardive dystonia. *Neurology, 73*(1), 53.

10. Isaias, I. U., Volkmann, J., Kupsch, A., Burgunder, J. M., Ostrem, J. L., Alterman, R. L., Tagliati, M. (2011). Factors predicting protracted improvement after pallidal DBS for primary dystonia: the role of age and disease duration. *J Neurol, 258*(8), 1469.

11. Kim, J. P., Chang, W. S., & Chang, J. W. (2011). Treatment of secondary dystonia with a combined stereotactic procedure: long-term surgical outcomes. *Acta Neurochir (Wien), 153*(12), 2319.

12. Kiss, Z. H., Doig-Beyaert, K., Eliasziw, M., Tsui, J., Haffenden, A., & Suchowersky, O. (2007). The Canadian multicentre study of deep brain stimulation for cervical dystonia. *Brain, 130*(Pt 11), 2879-2886

13. Krause, M., Fogel, W., Kloss, M., Rasche, D., Volkmann, J., & Tronnier, V. (2004). Pallidal stimulation for dystonia. *Neurosurgery, 55*(6), 1361.

14. Krause, M., Fogel, W., Tronnier, V., Pohle, S., Hortnagel, K., Thyen, U., & Volkmann, J. (2006). Long-term benefit to pallidal deep brain stimulation in a case of dystonia secondary to pantothenate kinase-associated neurodegeneration. *Mov Disord, 21*(12), 2255.

15. Kupsch, A., Benecke, R., Muller, J., Trottenberg, T., Schneider, G. H., Poewe, W., Volkmann, J. (2006). Pallidal deep-brain stimulation in primary generalized or segmental dystonia. *N Engl J Med, 355*(19), 1978.

16. Limousin, P., Pollak, P., Benazzouz, A., Hoffmann, D., Le Bas, J. F., Broussolle, E., Benabid, A. L. (1995). Effect of parkinsonian signs and symptoms of bilateral subthalamic nucleus stimulation. *Lancet, 345*(8942), 91.

17. Lumsden, D. E., Kaminska, M., Gimeno, H., Tustin, K., Baker, L., Perides, S., Lin, J. P. (2013). Proportion of life lived with dystonia inversely correlates with response to pallidal deep brain stimulation in both primary and secondary childhood dystonia. *Dev Med Child Neurol, 55*(6), 567.

18. Markun, L. C., Starr, P. A., Air, E. L., Marks, W. J., Jr., Volz, M. M., & Ostrem, J. L. (2012). Shorter disease duration correlates with improved long-term deep brain stimulation outcomes in young-onset DYT1 dystonia. *Neurosurgery, 71*(2), 325.

19. Nasreddine, Z. S., Phillips, N. A., Bedirian, V., Charbonneau, S., Whitehead, V., Collin, I., Chertkow, H. (2005). The Montreal Cognitive Assessment, MoCA: a brief screening tool for mild cognitive impairment. *J Am Geriatr Soc, 53*(4), 695.

20. Okun, M. S., Gallo, B. V., Mandybur, G., Jagid, J., Foote, K. D., Revilla, F. J., Tagliati, M. (2012). Subthalamic deep brain stimulation with a constant-current device in Parkinson's disease: an open-label randomised controlled trial. *Lancet Neurol, 11*(2), 140.

21. Pretto, T. E., Dalvi, A., Kang, U. J., & Penn, R. D. (2008). A prospective blinded evaluation of deep brain stimulation for the treatment of secondary dystonia and primary torticollis syndromes. *J Neurosurg, 109*(3), 405.

22. Sano, M., Zhu, C. W., Whitehouse, P. J., Edland, S., Jin, S., Ernstrom, K., Ferris, S. H. (2006). ADCS Prevention Instrument Project: pharmacoeconomics: assessing health-related resource use among healthy elderly. *Alzheimer Dis Assoc Disord, 20*(4 Suppl 3), S191.

23. Schjerling, L., Hjermind, L. E., Jespersen, B., Madsen, F. F., Brennum, J., Jensen, S. R., Karlsborg, M. (2013). A randomized double-blind crossover trial comparing subthalamic and pallidal deep brain stimulation for dystonia. *J Neurosurg, 119*(6), 1537.

24. Schuepbach, W. M., Rau, J., Knudsen, K., Volkmann, J., Krack, P., Timmermann, L., Deuschl, G. (2013). Neurostimulation for Parkinson's disease with early motor complications. *N Engl J Med, 368*(7), 610.

25. Valldeoriola, F., Morsi, O., Tolosa, E., Rumia, J., Marti, M. J., & Martinez-Martin, P. (2007). Prospective comparative study on cost-effectiveness of subthalamic stimulation and best medical treatment in advanced Parkinson's disease. *Mov Disord, 22*(15), 2183.

26. Vidailhet, M., Yelnik, J., Lagrange, C., Fraix, V., Grabli, D., Thobois, S., Pollak, P. (2009). Bilateral pallidal deep brain stimulation for the treatment of patients with dystonia-choreoathetosis cerebral palsy: a prospective pilot study. *Lancet Neurol, 8*(8), 709.

27. Volkmann, J., Wolters, A., Kupsch, A., Muller, J., Kuhn, A. A., Schneider, G. H., Benecke, R. (2012). Pallidal deep brain stimulation in patients with primary generalised or segmental dystonia: 5-year follow-up of a randomised trial. *Lancet Neurol, 11*(12), 1029.

28. Weaver, F. M., Follett, K., Stern, M., Hur, K., Harris, C., Marks, W. J., Jr., Huang, G. D. (2009). Bilateral deep brain stimulation vs best medical therapy for patients with advanced Parkinson disease: a randomized controlled trial. *JAMA, 301*(1), 63.

26. Abbreviations and Definitions

26.1. *Abbreviations*

The abbreviations used in the document are provided below.

Table 26-1: Abbreviations

Abbreviation/Acronym	Term
ADE	Adverse Device Effect
ADL	Activities of Daily Living
AE	Adverse Event
BFMDRS	Burke-Fahn-Marsden Dystonia Rating Scale
BSC	Boston Scientific Corporation
CCG	Case Report Form Completion Guidelines
CGI-C	Clinical Global Impression of Change
CI	Confidence Interval
CRF	Case Report Form
CT	Computerized Tomography Scan
CVA	Cerebrovascular Accident

Abbreviation/Acronym	Term
DBS	Deep Brain Stimulation
DFU	Directions for Use
€	Euro
EDC	Electronic Data Capture
ESAP	End of Study Action Plan
FCC	Federal Communications Commission
FCE	Field Clinical Engineer
FDA	Food and Drug Administration
GCP	Good Clinical Practice
GDS	Global Dystonia Rating Scale
GPi	Globus pallidus interna
HCP	Health Care Professional
HIPAA	Health Insurance Portability and Accountability Act
HRQoL	Health-related quality of life
ITT	Intent-To-Treat
ICF	Informed Consent Form
ICH	International Conference on Harmonization
IDE	Investigational Device Exception
IPG	Implantable Pulse Generator
IRB	Institutional Review Board
ISO	International Organization for Standardization
MoCA	Montreal Cognitive Assessment
MRI	Magnetic Resonance Imaging
PMA	Pre-Market Approval
RF	Radiofrequency
RUI	Resource Utilization Inventory

Abbreviation/Acronym	Term
SADE	Serious Adverse Device Effect
SAE	Serious Adverse Event
SE	Schwab and England Scale
SF-36v2	Medical Outcomes Study 36-item functional health survey version 2 - to be used in subjects age 14 years and older
SF-10v2	Medical Outcomes Study 10-item functional health survey version 2 - to be used in subjects younger than 18 years
STN	Subthalamic Nucleus
SWT	Satisfaction with Treatment
SureTek™	Trade name for BSC Burr Hole Cover
TWSTRS	Toronto Western Spasmodic Torticollis Rating Scale
USADE	Unanticipated Serious Adverse Device Effect

26.2. *Definitions*

The terms used in the document are defined below.

Table 26-2: Definitions

Term	Definition
Activation	The process of turning on the implantable pulse generator (IPG) for the first time after implant and the programming of stimulation parameters
CE Mark	The CE mark, or formerly EC mark, is a mandatory conformity marking for certain products sold within the European Economic Area (EEA) since 1985.
Enrollment	A subject is considered to be enrolled as a research subject in the study after informed consent is obtained

Term	Definition
End of Study Action Plan	Defines the actions to be taken when the subject reaches the end of their study participation
Monopolar review	A process where the therapeutic window for each contact is determined, i.e., measurement of efficacy and side effect thresholds
Source Data	All information in original records of clinical findings, observations, or other activities in a clinical investigation, necessary for the reconstruction and evaluation of the clinical investigation.
Source Document	Printed, optical or electronic document containing source data. Examples: Hospital records, laboratory notes, device accountability records, photographic negatives, radiographs, records kept at the investigation site, at the laboratories and at the medico-technical departments involved in the clinical investigation.
Vercise ™	The Vercise™ DBS system manufactured by Boston Scientific Neuromodulation
